# Research Review: On the (mis)use of puberty data in the ABCD Study® – a systematic review, problem illustration, and path forward

**DOI:** 10.1111/jcpp.70035

**Published:** 2025-08-25

**Authors:** Adriene M. Beltz, Holly Pham, Tristin Smith, Esmeralda Hidalgo‐Lopez, Hannah Becker, Christel M. Portengen, Mary M. Heitzeg, Chelsea Kaplan, Sheri A. Berenbaum

**Affiliations:** ^1^ Department of Psychology University of Michigan Ann Arbor MI USA; ^2^ Department of Psychology The Pennsylvania State University University Park PA USA; ^3^ Department of Anesthesiology, Chronic Pain and Fatigue Research Center University of Michigan Medical School Ann Arbor MI USA; ^4^ Department of Psychiatry Michigan Medicine Ann Arbor MI USA; ^5^ Neuroscience Graduate Program University of Michigan Ann Arbor MI USA; ^6^ Department of Pediatrics The Pennsylvania State University, Penn College of Medicine Hershey PA USA

**Keywords:** Adolescence, Adolescent Brain Cognitive Development Study®, measurement, Pubertal Development Scale, pubertal status, sex differences, systematic review

## Abstract

**Background:**

The Adolescent Brain Cognitive Development^SM^ (ABCD) Study® has significant potential to reveal the nature, causes, context, and consequences of pubertal development in diverse American youth. Optimal use of the data requires thoughtful consideration of puberty: how it is likely to affect psychological and neural development, and its measurement. We examined how ABCD puberty data have been used, and the relative advantages of two measures derived from the Pubertal Development Scale: the categorical measure provided in data releases and a continuous measure widely used outside ABCD.

**Methods:**

First, we conducted a review of published studies using ABCD puberty data through December 2024, following PRISMA guidelines. Two independent raters coded the studies for key features. Second, we used data from ABCD baseline and the Year 1 follow‐up to empirically compare the categorical and continuous measures in descriptives, reliability, sex differences, twin similarities, and examine correspondence.

**Results:**

Systematic review results from 190 reports showed that more studies considered puberty as a covariate (72%) than a variable of interest (28%), with 44% using the categorical measure from data releases and another 28% providing insufficient information to determine measurement. When puberty was a focus, there was variability in the use of youth versus parent‐report and approach to missing data, and little attention to sex differences. Results from the empirical comparison showed advantages of the continuous over the categorical measure in data availability, developmental distributions, and reliability; they also confirmed the limited utility of youth‐report in early puberty.

**Conclusions:**

Results have crucial implications for the use of ABCD puberty data, especially early assessments. They highlight the complexity of studying pubertal influences on adolescent development and emphasize measurement. Attention to these issues will maximize the potential of ABCD to rigorously delineate the role of puberty in brain and behavioral development and to ultimately promote youth well‐being.

## Introduction

The Adolescent Brain Cognitive Development^SM^ (ABCD) Study® is the largest long‐term study of brain development and adolescent health in the United States (11,868 youth recruited across 21 research sites, enriched for twin pairs), with the goal to ‘determine how childhood experiences … interact with each other and with a child's changing biology to affect brain development and social, behavioral, academic, health, and other outcomes’ (ABCD Study, 2024). Annual assessments of puberty starting at ages 9–10 years using an established measure have the potential to enrich understanding of adolescent development, clarifying how advancing age reflects biological changes associated with reproductive maturity, and how pubertal processes shape and are shaped by experiences in ways that change the brain and behavior. The value of the data depends on thoughtful consideration of the complexity of pubertal development and the use of variables that accurately reflect puberty in its behavioral and neural significance. Current practices, however, may undermine that potential and contribute to the dissemination of misinformation about the correlates of puberty.

The goal of this article is to evaluate the conceptualization of puberty in ABCD, as reflected in early study assessments and in detailed analyses comparing different measures derived from ABCD data. Issues are discussed; recommendations are offered for using the data to maximize the potential of ABCD to address open questions about the role of puberty in adolescent development.

## The importance of puberty for adolescent development

Puberty contributes to adolescent psychological development in several ways, and is critical to assess in studies of developmental psychopathology (Klump, 2022). *Pubertal status* – how far along a youth is in pubertal development – has direct effects via hormones acting on the brain and indirect effects via personal and social responses to physical changes; progression through puberty is often described by Tanner stages (Tanner, 1978). Pubertal processes are hypothesized to induce both normative changes in behaviors (e.g. risk‐taking) and to trigger psychopathology in some youth (e.g. increased incidence of depression in female youth; reviewed in Dorn & Beltz, 2023). *Pubertal timing* – a youth's pubertal status relative to same‐sex others – has been shown to influence several behaviors, with most work showing behavioral risk for female adolescents who mature early, but increasing evidence for risk from both early and late maturation in both sexes, and some risks persisting into adulthood (Dorn & Beltz, 2023). During adolescence, when development is ongoing, it is difficult to differentiate pubertal status from pubertal timing. For example, a female 9‐year‐old with moderate breast development has both advanced status and early timing, according to this secondary sexual characteristic. *Pubertal tempo* – the speed with which an adolescent proceeds through the different stages of puberty – has not been well‐studied; there is no consensus on its operationalization, which benefits from true longitudinal data.

ABCD provides a unique opportunity to answer important questions about correlates of pubertal status, timing, and tempo. Annual assessments will ultimately enable optimal measurement of pubertal timing and tempo by leveraging longitudinal assessments (see Berenbaum, Beltz, & Corley, 2015; Mendle, Beltz, Carter, & Dorn, 2019), and the differentiation of pubertal timing and status. The sample is large and diverse, providing high power and generalizability and the ability to conduct cross‐validated analyses by site (Karcher & Barch, 2021).

## Describing the measurement of puberty in ABCD

At each ABCD assessment, data are obtained on youth's current pubertal development using two measures of status. The first, salivary hormones, has limitations. Only one sample was obtained during a test session scheduled between 7 AM and 7 PM (Herting et al., 2021); the observed data do not necessarily reflect this (e.g. the lag between awakening and sample collection ranges from 0 to ~18 hrs at baseline and 0 to ~16 hrs at the Year 1 follow‐up, and sample collection precedes awakening in some cases). There was also minimal control of factors that influence hormone levels, including diurnal rhythms, menstrual cycle, and exercise (see Cheng et al., 2021; Uban et al., 2018). This measurement noise underestimates effects even in large samples, potentially resulting in Type II error if the investigated effects are small, as are most behavioral effects of puberty and in the ABCD Study (see Baams, Dubas, Overbeek, & van Aken, 2015; Loken & Gelman, 2017; Owens et al., 2021; Ullsperger & Nikolas, 2017). Moreover, the reliability and validity of the assays, especially estradiol (Rosner, Hankinson, Sluss, Vesper, & Wierman, 2013), have not been established in this or comparable samples of youth in early pubertal stages who may have low hormone levels. It may be possible to use data from selected participants (Cheng et al., 2021; Herting et al., 2021) but even with perfect measurement, a single sample may not reflect pubertal development. A cross‐sectional sample will be confounded by other neuroendocrine processes reflected in salivary hormones, such as within‐person variations in menstrual cycle phase, diurnal rhythms, and environmental circumstances (e.g. food intake; Ahn et al., 2011; Matchock, Dorn, & Susman, 2007; Schwartz et al., 2019). For further discussion, see Berenbaum et al. (2015) and Dorn, Dahl, Woodward, & Biro (2006).

Thus, our review focuses on ABCD's second measure of status: the Pubertal Development Scale (Petersen, Crockett, Richards, & Boxer, 1988). The youth and a caregiver separately rate the youth's physical development of secondary sexual characteristics, including body hair, skin changes, and growth spurt in both sexes, facial hair and deepening voice in male youth, and breast development and menarche in female youth. Menarche is reported as *absent* (coded 1) or *completed* (coded 4); other items are rated on a full 4‐point scale (1: *no development* to 4: *completed*). The PDS is widely used and generally accepted as a broad estimate of development, especially when used longitudinally, but scores must be considered in light of some limitations, including greater inaccuracies at some stages than others, apparent regressions in development, and inconsistencies between youth and caregiver report (Berenbaum et al., 2015; Dorn et al., 2006; Shirtcliff, Dahl, & Pollak, 2009).

Following the work introducing the PDS (Petersen et al., 1988), most studies average the five items, resulting in scores ranging from 1 to 4 – hereafter referred to as the *PDS continuous* measure. This common measure is noted in key papers describing ABCD puberty data (Cheng et al., 2021; Herting et al., 2021), and can be computed from the data releases. Nevertheless, it is not the summary score provided in ABCD releases.

Instead, the ABCD summary score included in releases is a 5‐point categorical score – hereafter referred to as the *ABCD categorical* measure – representing pubertal stage (pre‐, early‐, mid‐, late‐ or post‐puberty) based on cut points in the sum of three PDS items (male youth: body hair, facial hair, and voice changes; female youth: body hair and breast development, with menarche required for a late‐puberty score; see Appendix S1). This score is described in an ABCD Study consortium article (Herting et al., 2021) and seems to be derived from an unpublished script referenced in early studies employing the PDS (Carskadon & Acebo, 1993; Petersen et al., 1988); see also Appendix S2.

The ABCD categorical measure is problematic for several reasons. First, no rationale or empirical support is provided for highlighting this measure rather than the continuous measure. Scoring information for the categorical measure was not provided, and details are still not published but circulate informally; until now, this measure has rarely been used. It may be recommended because the categories are thought to map onto Tanner stages, but there is no published empirical basis for the cut points, and other data indicate poor correspondence between the five categories and Tanner stages (Koopman‐Verhoeff, Gredvig‐Ardito, Barker, Saletin, & Carskadon, 2020). A different attempt to use the PDS to approximate Tanner stages for adrenarche (maturation of the adrenal glands) and gonadarche (maturation of the gonads) has been more widely used than the ABCD categorical measure (Shirtcliff et al., 2009), but it is based on a cross‐sectional sample of 160 early adolescents and has not been validated in large samples spanning pubertal development. Second, the selection and use of specific features are problematic: Menarche is emphasized in assigning female youth to categories, and important social signals of growth spurt and skin changes are omitted, challenging the validity and reliability of the categorical measure as a holistic reflection of pubertal development. Third, statistical problems result in lost information: Missing data on a single item prevent the calculation of a score (items are summed, and scores are not prorated), so youth missing a single item are excluded (which may result in selective exclusions). Fourth, the categorical measure is often treated as continuous in analyses (assigned scores 1–5).

## Evaluating the measurement of puberty in ABCD

We evaluated the extent to which inferences about puberty and adolescent development might be distorted by the use of the ABCD categorical measure provided in the data releases. Our evaluation included two parts: a systematic review of the uses of ABCD puberty data; and an empirical comparison of the ABCD categorical and PDS continuous measures on basic descriptive information, reliability, sex differences, twin similarities, and their correspondence to each other. These two parts inform each other, as empirical differences between the measures could be mitigated or exacerbated depending upon how the measures are used in the extant corpus of puberty investigations leveraging ABCD Study data.

### Systematic review: Tallying the use of ABCD puberty data

We conducted a systematic review of the ways that the PDS data from the ABCD Study have been used, including reports published through December 31, 2024, using the baseline and Year 1 follow‐up data releases because enough time had elapsed to provide a nearly comprehensive, representative number of publications while limiting methodological complexities surrounding longitudinal data.

**Figure 1:**
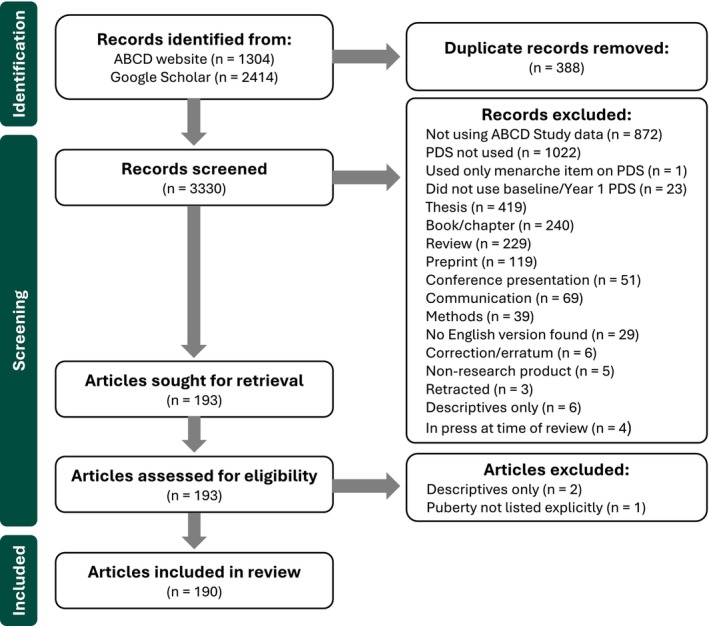
Articles included in the systematic review, following Preferred Reporting Items for Systematic Reviews and Meta‐Analyses (PRISMA) guidelines. The systematic review included peer‐reviewed empirical reports of puberty (using the Pubertal Development Scale, PDS) in the ABCD Study baseline or Year 1 follow‐up data, published through December 31, 2024. Some reports are based on overlapping data beyond the measure of puberty, and descriptive reports only used the PDS to characterize the sample

The procedure for identifying studies for review is described in Figure 1; studies included are listed in Appendix S3. Following the Preferred Reporting Items for Systematic Reviews and Meta‐Analyses (PRISMA) guidelines (Page et al., 2021), studies were identified through the ABCD publications archive (https://abcdstudy.org/publications/; ABCD Study, 2024) using the search term ‘PUBERT’ or through GoogleScholar using the terms ‘ABCD & PUBERT*’. Duplicate records were removed, and abstracts were screened to determine an article's relevance; most excluded articles did not use ABCD data or the PDS, or were not peer‐reviewed empirical works. The remaining 193 articles underwent full review, uncovering three additional exclusions. Coding was conducted by two independent raters, and discrepancies were reconciled by a third rater.

All studies were reliably coded for: (a) puberty as a variable of interest versus covariate; (b) whether the categorical measure provided in ABCD releases was used; and (c) aspect of puberty studied: status or timing. Studies with puberty as a variable of interest were also coded for: (d) source of report: parent or youth; (e) handling of missing data; (f) consideration of sex differences; and (g) additional use of hormone data, with consistency of findings across hormones and reports of physical development.

#### All studies

**Figure 2:**
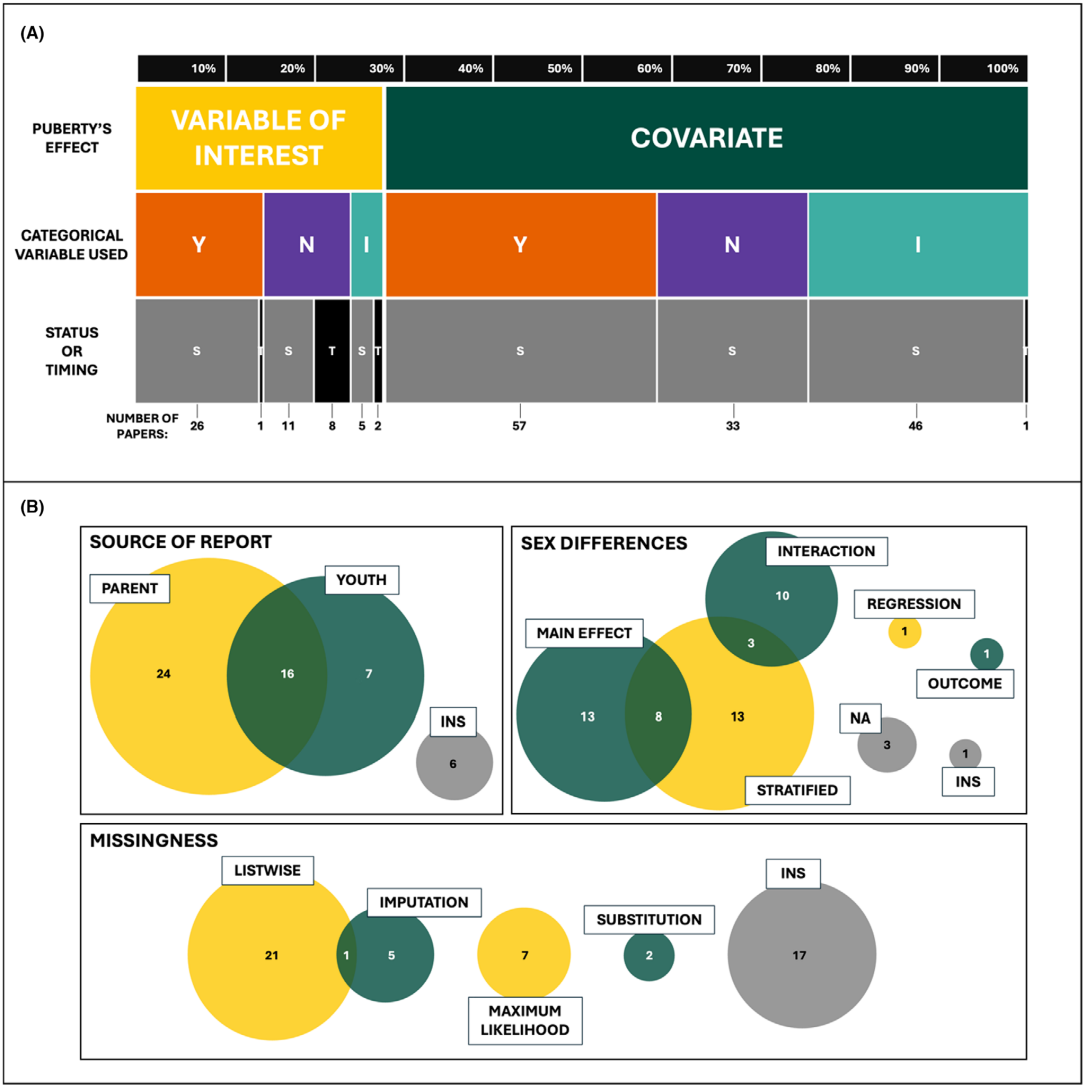
Systematic review results. Panel A shows results from the systematic review of the 190 total empirical reports published using Pubertal Development Scale (PDS) data from ABCD Study baseline or Year 1 follow‐up: if puberty was included as a variable of interest in analyses (yellow) or considered a covariate (green), if the ABCD categorical variable was used (orange) or not (purple) or if there was insufficient information to determine this (teal), and if pubertal status (gray) or timing (black) was examined. The black bar at the top shows the proportion of reviewed articles in 10% increments. Panel B shows results focused on the 53 reports that examined puberty's effect as a variable of interest: if parent (yellow) or youth (green) PDS report was used, how sex differences were addressed, and how missingness of PDS items was addressed. Some of these reports used a combination of approaches, which is shown through overlapping circles; other reports did not provide sufficient information to determine an approach (gray). Y = yes; N = no; I/INS = insufficient information to determine; S = pubertal status; T = pubertal timing; NA = not applicable

Results of the review are shown in Figure 2A for a–c coding conducted for all studies. The median bias‐ and prevalence‐adjusted kappa was .87.

##### Puberty as a variable of interest or covariate

The focus of most studies was not on puberty itself, but on covarying puberty in examining a variety of other research questions, ranging from brain structure and function to mental health and cognition to identity and context (Figure 2A, top row). In these studies (72% of the total), there was little appreciation of the limitations of covarying, which assumes that puberty is linearly related to the outcome(s) of interest and that it does not interact with other covariates or the predictor(s) in affecting an outcome. Furthermore, the systematic review suggested little attention was paid to measurement, with studies investigating puberty as a covariate generally containing fewer details (e.g. reporter, missing data) than puberty‐focused studies.

##### Puberty measure used

Specific information about the measure used was more likely to be provided in reports with puberty as a variable of interest than in those with puberty as a covariate, with 13% vs. 34% of articles in the respective categories providing insufficient information to determine what measure was used (Figure 2A, middle row). Half of the former used the ABCD categorical measure (and varied in whether it was considered categorical or continuous in analyses). Justification was rarely provided for the puberty measure used; perhaps reflecting assumptions that the ABCD categorical measure was optimal because it was included in data releases.

##### Pubertal status versus timing

Most studies examined pubertal status, but a minority (6%) examined pubertal timing, with most using age‐regressed pubertal status residuals in analyses (Figure 2A, bottom row). During puberty (when data collection occurred) there is confounding of pubertal status and timing, as noted above. Therefore, a single measure of development (either the ABCD categorical measure or the PDS continuous measure) is usually used to index pubertal status, as elaborated below.

#### Studies with puberty as a variable of interest

Results are shown in Figure 2B for d–f coding conducted for studies considering puberty as a variable of interest. The median bias‐ and prevalence‐adjusted kappa was .64.

##### Source of report

There was considerable variability in the source of reports of youth physical development (Figure 2B, top left). Although more used parent‐report (45%) than youth‐report (13%), a significant number took hybrid approaches, including replacing a missing youth‐reported item with the same parent‐reported item or vice versa, averaging reports, and replacing an average with a single report if the other report was missing; some papers (11%) did not provide enough information to classify the source of the report.

##### Missing data

A plurality of studies (40%) excluded youth with missing scores, either pairwise or listwise, and a similar percentage (32%) did not describe missing data approaches; other approaches involved using different reporters for different youth, as mentioned above (Figure 2B, bottom). About a quarter of studies (23%) used contemporary, structured approaches to missing data, including imputation or maximum likelihood estimation (Lang & Little, 2018). Different approaches to missing data have implications that were generally not discussed, including selective exclusion (e.g. of female youth with early development). As reported below, data are more likely to be missing for the ABCD categorical than for the PDS continuous measure, raising additional concerns about unsystematic solutions to missing data.

##### Sex differences

Given established sex differences in pubertal development (Styne & Grumbach, 2016), it is surprising that they were often ignored (Figure 2B, top right). Often, male and female youth were combined, and analyses focused on main effects of puberty while treating sex as a covariate (40%). Some studies (25%) considered sex as a moderator that might interact with other predictors in affecting outcomes, among a variety of other approaches; for instance, a few studies employed single‐sex samples, and one regressed puberty on sex to account for differences.

The failure to attend to sex differences is a major problem because of differences in pubertal timing and in the neuroendocrine processes of puberty (Beltz, Beery, & Becker, 2019). Combining the sexes confounds puberty and sex, and may lead to suppression effects when examining other biopsychosocial processes, as highly correlated predictors (e.g. puberty and sex in early adolescence) may relate to outcomes in ways that vary in both magnitude and direction compared with predictors’ unique relations to outcomes (Ludlow & Klein, 2014).

One report illustrates the value of examining the effects of sex because sex differences were seen in the distributions of ABCD variables (Palmer et al., 2021). In studies included in our systematic review, interactions between sex and puberty on brain and behavior were not always observed (perhaps for methodological reasons, including the underrepresentation of male adolescents with advanced pubertal status), but were examined in a few (Holm et al., 2023; Loso et al., 2021; Gadassi Polack, Mollick, Keren, Joormann, & Watts, 2023; Serio, Kohler, Ye, Lichenstein, & Yip, 2022; Vijayakumar, Whittle, & Silk, 2023).

##### Puberty measured with reports and hormones

A minority of studies (25%) used both hormonal and survey measures (generally the ABCD categorical measure). Of those papers, 38% (5 of 13) had consistent findings across measurement types; most others had disparate patterns of results. These inconsistent findings combined with the concerns noted above about the limitations of a single sample of saliva urge caution in using the hormone data.

#### Other considerations

Our review also highlighted other issues that are important in using the puberty data, such as neglecting to consider the complexity of the ABCD Study and to adjust analyses accordingly (Saragosa‐Harris et al., 2022). Two issues are particularly relevant: Family dependencies resulting from multiple siblings, including twins, participating in the study need to be considered, especially given genetic influences on pubertal development (Pham, Beltz, Corley, & Berenbaum, 2022); sampling variations across study sites result in confounding of pubertal measures with study site (e.g. given race and ethnic differences in pubertal development; Deardorff, Hoyt, Carter, & Shirtcliff, 2019; Mendle et al., 2019).

#### Summary

Consistent with data releases, the ABCD categorical measure was widely used. The lack of attention given to measure selection in the reviewed reports was apparent in several ways, especially in the number of studies that provided insufficient information about the measure and the ubiquitous ad hoc approaches to missing data. Use of parent‐ versus youth‐reports also varied across studies. Important conceptual issues were often not considered, including assumptions involved in using puberty or sex as covariates, limitations of the hormone measures, and the importance of different aspects of puberty for brain and behavioral development. In essence, there seemed to be little consideration of the implications of the choice of measure.

### Empirical comparison: Measures of pubertal status in ABCD data

To demonstrate how measurement might affect inferences about puberty's role in adolescent development, we compared the released ABCD categorical measure with the widely used PDS continuous measure (as revealed in the extant literature beyond ABCD). We focused on data from the assessments at baseline and the Year 1 follow‐up, and started with both youth self‐ and parent‐reports. Data used here were obtained from participants who provided informed assent and parental informed consent, overseen by the Institutional Review Boards of each study site. Statistics reported below are based on data from only one sibling per family (see Appendix S4), whereas illustrative plots often include all participants. Participant numbers vary across analyses, as described.

#### Missing data

**Figure 3:**
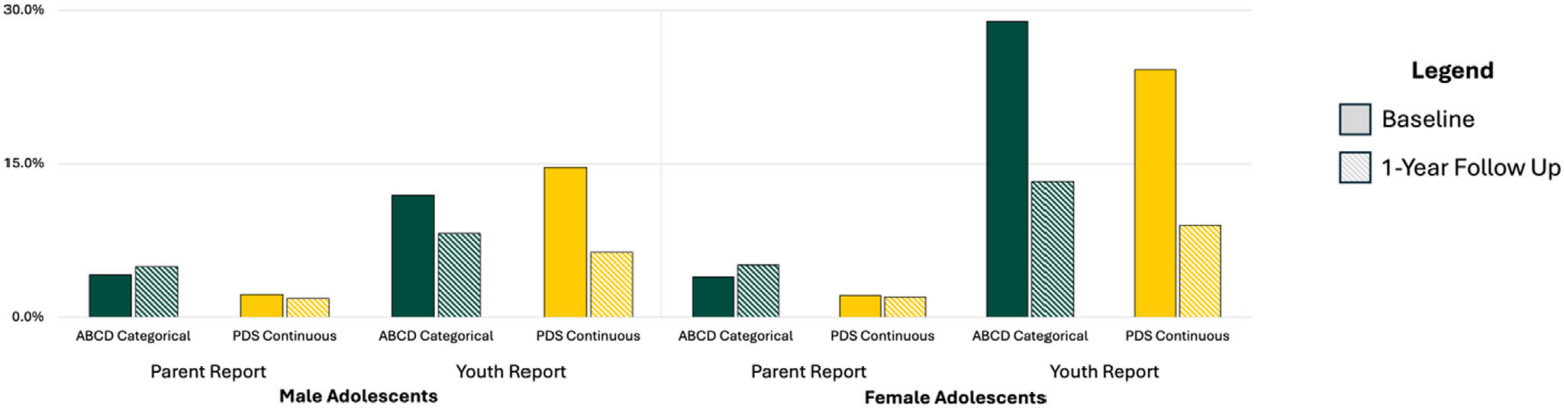
Percent of missing data by puberty measure, timepoint, reporter, and sex. For the ABCD categorical measure (green), missing data occur when at least one PDS item of three (for male adolescents: body hair, facial hair and voice changes; for female adolescents: body hair, breast development, and menarche) is missing, but the PDS continuous measure (yellow) is calculated if at least four of five PDS items are answered. Percentages are based on youth with all assessments and only one sibling per family (Males: Baseline *N* = 5,162, Year 1 follow‐up *N* = 4,882; Females: Baseline *N* = 4,645, Year 1 follow‐up *N* = 4,350)

The PDS continuous score (requiring at least four of five items, following Cohen, Cohen, West, & Aiken, 2013) is available for a larger and less selective sample than is the ABCD categorical score (requiring three of three items), as seen in Figure 3, reflecting the different approaches to item inclusion. This is the case for both sexes, assessment timepoints, and reporters (parent and youth). The only exception is male adolescents’ self‐report at baseline, with 11.9% missing for the ABCD categorical measure and 14.6% missing for the PDS continuous measure, likely indicating that some male youth only answered the three items used in the categorical measure. The figure also shows that missingness is especially high for self‐reports, particularly from female youth.

High rates of missing youth‐report data require cautious interpretation in empirical reports, as youth and parent‐reports on both measures systematically differ, and as categorical scores may not be missing at random. Composite scores in Table 1 show that male youth report more advanced development than do their parents on both measures and at both timepoints, but that female youth and parents only differ on the continuous measure, with parents reporting more advanced development. In all cases, though, development is more advanced according to the ABCD categorical than the PDS continuous measure.

**Table 1:** Composite score means (standard deviations) by puberty measure, timepoint, reporter, and sex

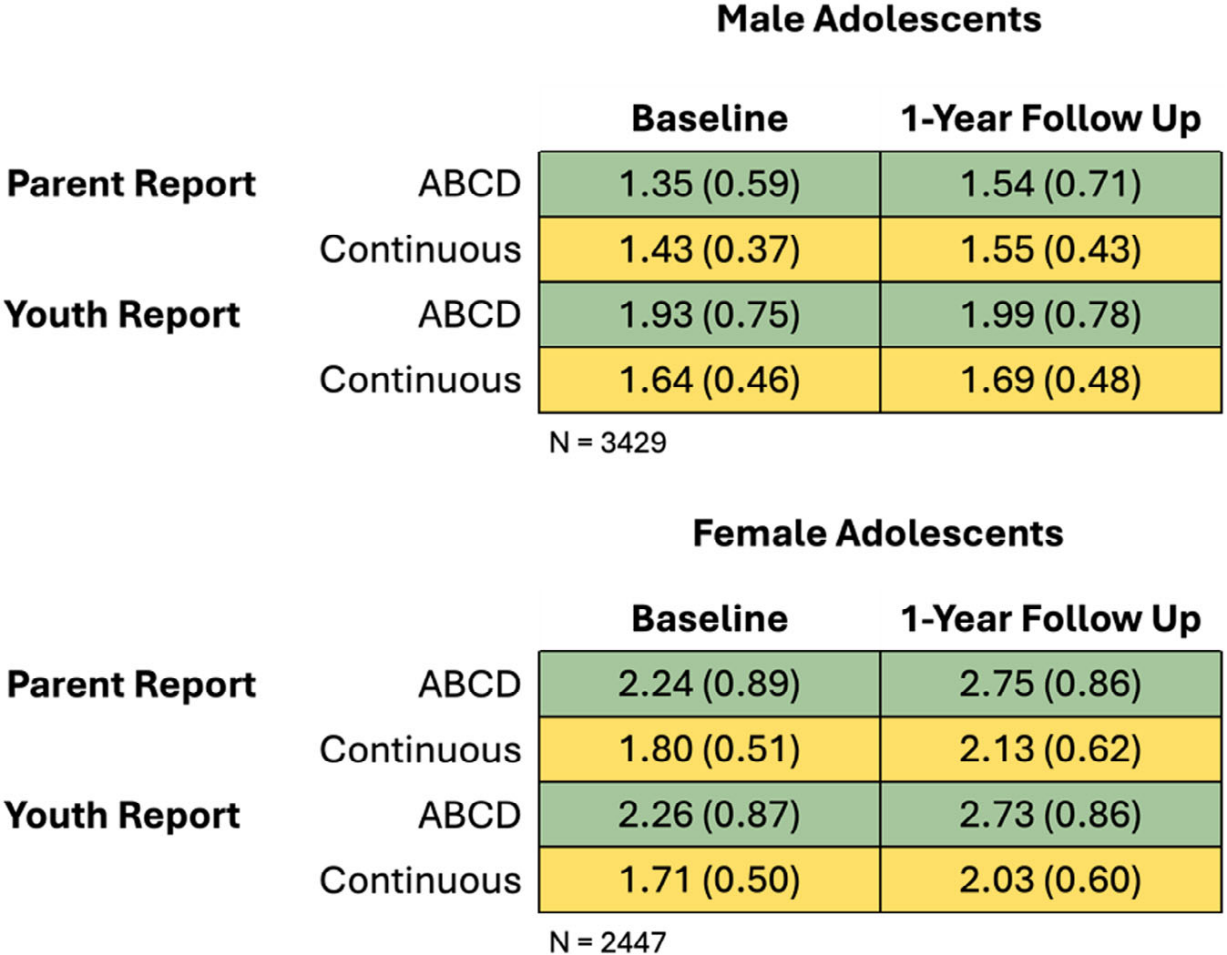

Data are shown only for individuals with relatively complete data (i.e., categorical and continuous measures from both self‐ and parent‐reports at both baseline and Year 1 follow‐up) to facilitate comparisons across cells.

Importantly, youth who have an ABCD categorical score may be selective; those missing the measure due to an unanswered item used in the sum score seem to have higher continuous PDS scores than those with ABCD categorical scores (e.g. at baseline, male adolescents: 1.57 vs. 1.44 and female adolescents: 1.96 vs. 1.77, respectively). This means that early maturers may be selectively missing ABCD categorical scores. This is particularly problematic for studies examining processes linked to early pubertal timing, including both potential antecedents (e.g. social determinants of health, such as early adversity, socioeconomic position, race, or ethnicity) and consequences (e.g. behavioral problems).

#### Measure distributions

**Figure 4:**
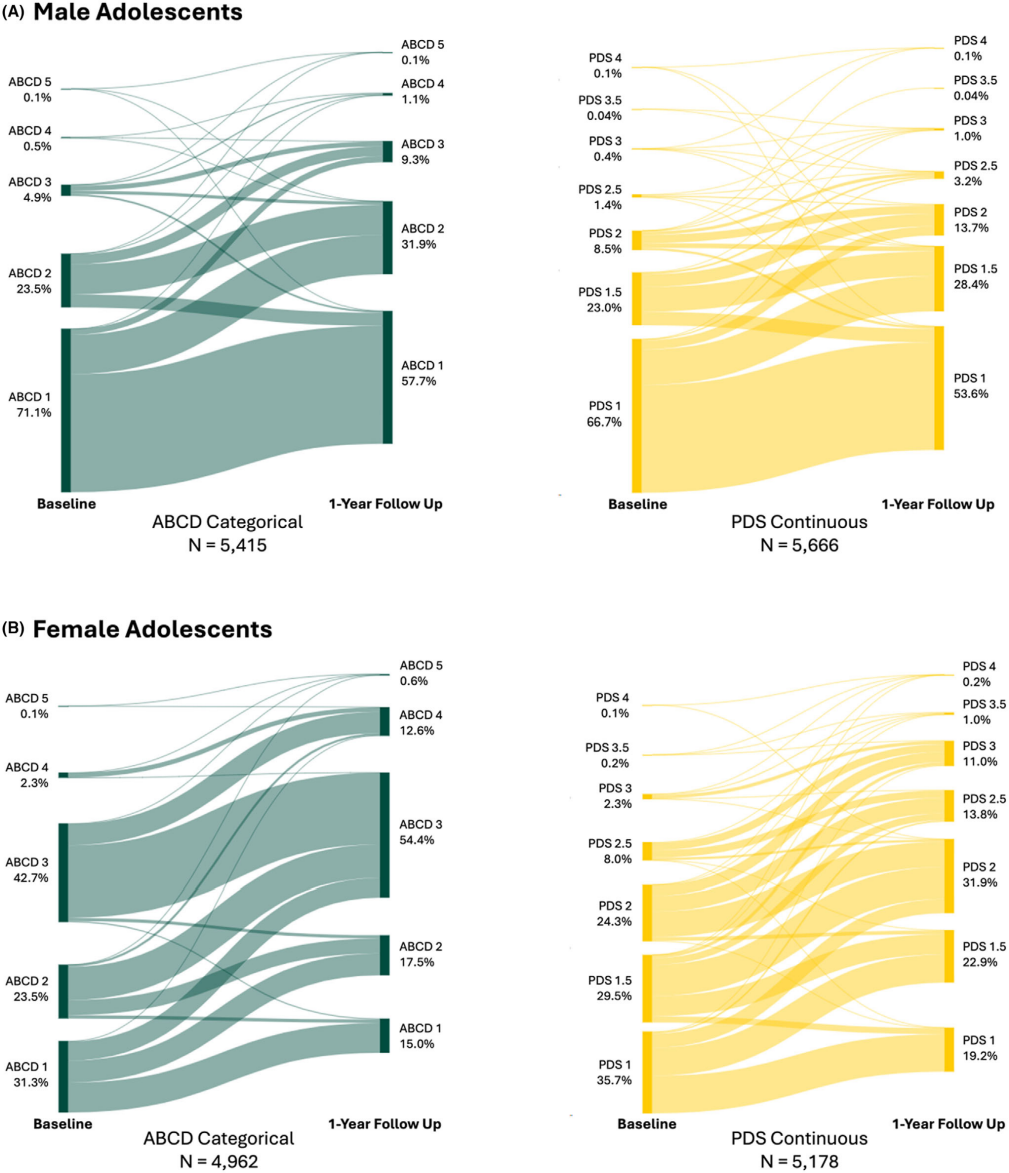
River plots of the distributions of parent‐reported pubertal development across timepoints for the ABCD categorical (in green) and PDS continuous (in yellow) measures, separately for male (A) and female (B) adolescents. Each plot shows the proportion of participants at baseline (left) and then the Year 1 follow‐up (right) with a given categorical or continuous measure score, with the PDS continuous measure binned in increments of 0.5. By following the ‘flow’ of the data from the left to the right, the proportion of youth who maintain the same score, progress to a higher score, or even regress to a lower score can be tracked; for instance, 35.7% of female adolescents had a PDS continuous score of 1 at baseline, but only 19.2% still had a continuous score of 1 a year later. Data show youth with measures at both timepoints

Figure 4 shows how the two measures reflect pubertal development across assessments for youth with parent‐report at both timepoints. (Data from youth self‐report are not shown given high rates of missingness.) Specifically, these river plots show the proportion of male (Figure 4A) and female (Figure 4B) adolescents with each ABCD categorical score and PDS continuous scores (binned in 0.5 increments) at baseline and Year 1 follow‐up, with line thickness reflecting how many youth transitioned from one score to another. For instance, 71.1% of male youth started with an ABCD categorical score of 1, with 57.7% still having a score of 1 at the Year 1 follow‐up; whereas only 31.3% of female youth had an ABCD categorical score of 1 at baseline and 15.0% had the same score a year later.

These plots provide several important insights. First, the assessment schedule has different effects for male and female youth owing to sex differences in pubertal onset. Most male youth have not developed beyond ‘pre‐puberty’ at Year 1 follow‐up, whereas two‐thirds of female youth were already in puberty at baseline, indicating that assessments started too late to capture pubertal onset for them. Second, female youth are considered to have much more advanced pubertal development on the ABCD categorical than on the PDS continuous measure. At Year 1 follow‐up, the categorical measure midpoint of 3 or higher is reached by 67.6% of female youth, but the PDS measure midpoint of 2.5 or higher is reached by only 26%. Thus, a stunning 2.5 times more female youth are classified as midpubertal or later by the ABCD categorical than by the PDS continuous measure. Third, the river plots show regressions in reported pubertal development, that is, individuals whose development is less advanced at Year 1 follow‐up than at baseline. This is not uncommon in puberty measurement (see Dorn et al., 2006) and seems to occur at similar rates for both measures.

#### Measure reliability

The following comparisons were based on youth with relatively complete data (i.e. those who had categorical and continuous youth‐ and parent‐report measures at baseline and Year 1 follow‐up) so samples are smaller than for analyses reported above. Specifically, assessments of reliability included youth with data for all puberty items at both timepoints for both reporters.

The PDS continuous measure was more reliable than the ABCD categorical measure. Median coefficient alpha for PDS continuous versus ABCD categorical, respectively, calculated across sex and timepoint (male youth: *n* = 2,110; female youth: *n* = 1,396) is: .70 versus .62 for parent‐report and .63 versus .54 for youth‐report. The comparatively higher reliabilities of the PDS continuous score may partly reflect the number of items comprising the scores (5 vs. 3). The higher reliabilities of parent‐ than youth‐report reinforce concerns with the latter.

#### Sex differences

Assessments of sex differences included youth who had both ABCD and PDS scores (and could be missing a single item). Sex differences were expected in both measures, given established differences in pubertal development (reviewed in Dorn & Beltz, 2023; Styne & Grumbach, 2016). The PDS continuous measure shows smaller sex differences than the ABCD categorical measure (male youth: *n* = 3,429; female youth: *n* = 2,447): respective Cohen's *d*s for parent‐reports are −0.83 versus −1.19 at baseline, and −1.09 versus −1.53 at Year 1 follow‐up. Male youth had similar scores on the two measures, but female youth had higher scores on the ABCD measure (as shown in Table 1). It is unclear which measure is a more accurate reflection of the sex difference in pubertal status, but the large sex difference in both measures emphasizes the confounding of sex and pubertal status in most studies reviewed. It also raises the question of the timing of menarche in the process of puberty, which is emphasized in categorical scores for female youth, who receive a score of 4 or more (out of 5) after menarche. This scoring minimizes the influence of the other two items in the categorical composite and reifies the common assertion that menarche occurs late in puberty (based on limited, decades‐old data from a small and selective sample; Marshall & Tanner, 1969).

#### Correspondence of measures

**Figure 5:**
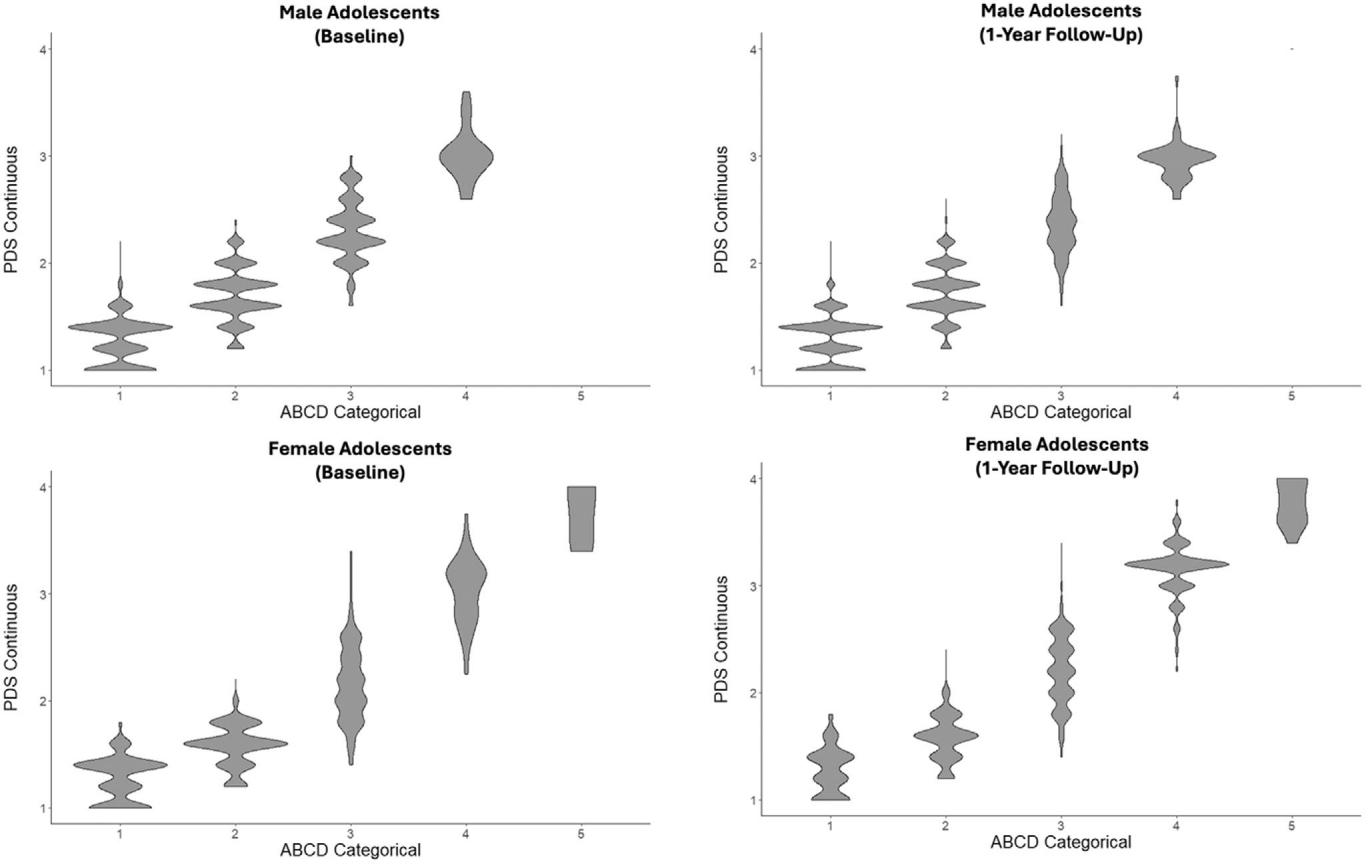
Violin plots of the correspondence between scores on the ABCD categorical (*x*‐axis) and PDS continuous (*y*‐axis) measures by timepoint and sex. The gray areas show distributions of overlapping scores for individuals with both measures at a timepoint and only one sibling per family (males: baseline *N* = 4,940, Year 1 follow‐up *N* = 4,635; females: baseline *N* = 4,451, Year 1 follow‐up *N* = 4,119)

Correlations of parent‐reported scores on the ABCD categorical and PDS continuous measures are high at both baseline and Year 1 follow‐up (male youth: *r* = .80 and .84; female youth: *r* = .82 and .86, respectively) using data from those with both scores at a timepoint. Nevertheless, as seen in Figure 5, violin plots crossing the scores for the two measures at baseline and Year 1 follow‐up show some discrepancies. In general, the correspondence between the measures progresses positively and linearly with development, but a male adolescent with a PDS continuous score of 2 could have an ABCD categorical score of 1, 2, or 3 at baseline or Year 1 follow‐up. Moreover, the emphasis on menarche in the ABCD categorical score is apparent in the mode at 4 at Year 1 follow‐up for female adolescents.

#### Twin similarities

The ABCD Study includes a subset of over 1,000 twin pairs. Variations in pubertal development are moderately to largely heritable (reviewed in Pham et al., 2022), so twin similarities on ABCD measures provide an index of validity. We examined similarities between twins who had both self‐ and parent‐report scores on both the ABCD categorical and PDS continuous measures at baseline and Year 1 follow‐up, separately by sex. As expected and shown in Table 2, similarities were higher among monozygotic twins than among same‐sex dizygotic twins for both male and female youth on both measures, reporters, and timepoints, except for female adolescents’ self‐reported ABCD categorical score. Again, youth self‐reports mostly showed low concordance.

**Table 2:** Twin correlations (*r*) by twin type, puberty measure, timepoint, reporter, and sex

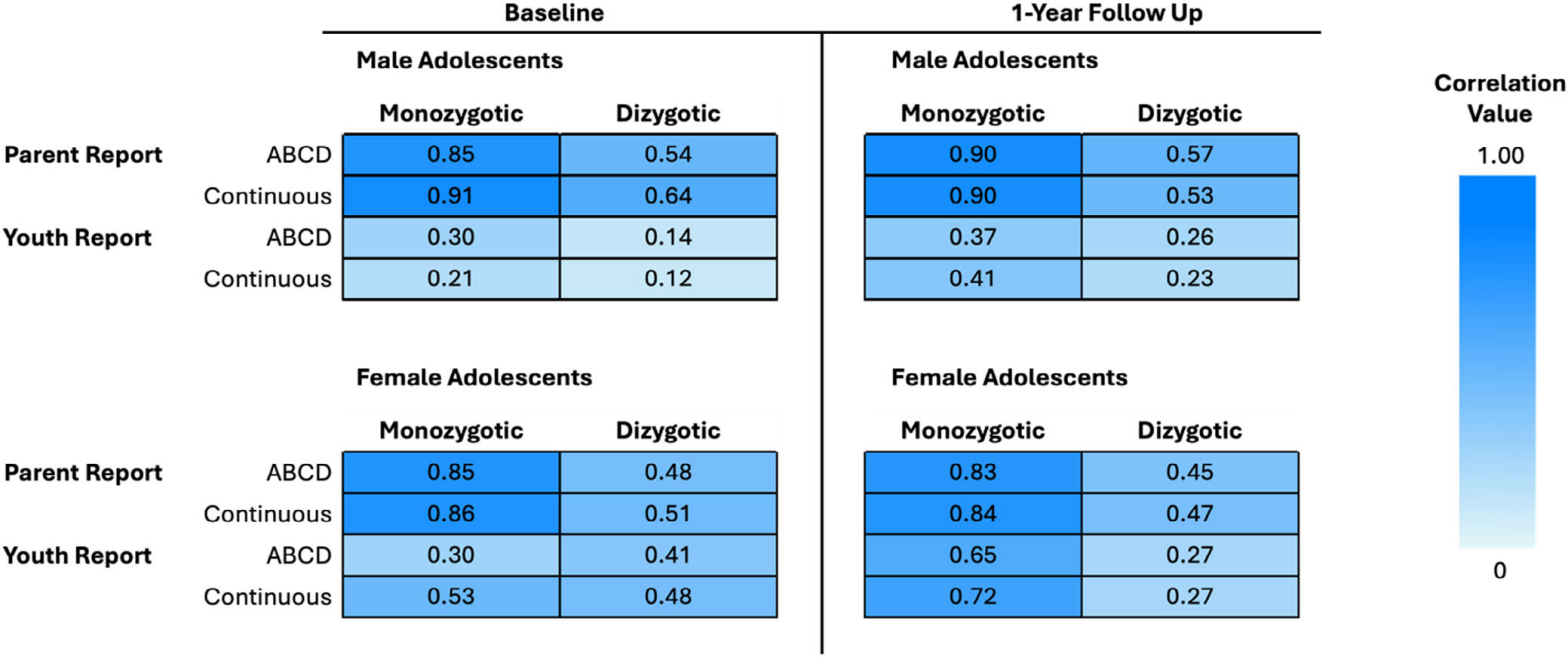

Data are shown only for individuals with all relevant data to facilitate comparisons across cells. For male adolescents, there were 109 monozygotic and 152 dizygotic pairs. For female adolescents, there were 89 monozygotic and 109 dizygotic pairs. All correlations are positive, and darker shades of blue reflect strong relations.

#### Summary

The empirical comparison generally shows the superiority of the PDS continuous measure over the ABCD categorical measure in terms of missing data (when following 80% item completion recommendations; Cohen et al., 2013), developmental distributions, and reliabilities in the early assessments of the ABCD Study. They also support other data on the limited utility of self‐report in pre‐ or early pubertal youth (reviewed in Dorn et al., 2006) and suggest that pubertal onset was missed for most female adolescents. Furthermore, the emphasis on menarche in the ABCD categorical score results in considerable discrepancies with the PDS continuous score, suggesting the need for more data on the timing of menarche in pubertal development, especially for modern cohorts.

## Maximizing benefits of ABCD puberty data

The availability of data from the ABCD Study® has expanded interest in addressing questions about pubertal influences on adolescent development and renewed focus on the need to specify how pubertal processes matter for the brain and behavior – using measures that adequately capture those processes (Dorn et al., 2006; Dorn & Beltz, 2023; Klump, 2022). Our systematic review of published studies and empirical comparison of measures highlights important points that need to be considered in work with ABCD data. Some concerns are specific to the use of the ABCD categorical score, but others apply generally to data based on youth self‐ or parent‐report of youth's physical development, and still others apply broadly to studying consequences of pubertal development – and are illustrated by studies using ABCD data.

In general, design and analysis need to be thoughtfully matched to the specific research questions being investigated, recognizing that puberty is not a unitary process and different aspects of puberty likely have differential significance. In this article, we only considered pubertal status, leveraging baseline and Year 1 follow‐up data published through December 2024 because articles using those data had sufficient time for peer review and publication as well as avoid complexities of longitudinal analyses. Youth, however, vary in several aspects of pubertal development, including not only status but also timing and tempo. Full longitudinal data across the developmental transition (as will eventually be available in ABCD) are necessary to disentangle the relative influences if the psychological implications of both early and late timing are of interest. The best estimates of pubertal timing come from random effects growth models that take advantage of multiple data points, validly adjust for missing data and apparent regressions in individual trajectories, and consider the trajectory of development (e.g. logistic; Beltz, Corley, Bricker, Wadsworth, & Berenbaum, 2014; Marceau, Ram, Houts, Grimm, & Susman, 2011). In early ABCD assessments, most youth are in the early‐to‐mid‐stages of puberty, so data best identify early maturing female adolescents and do not differentiate on‐time and late maturers nor provide meaningful variations in male adolescents given their comparatively later development. This might suffice for some research questions, in which case analyses and inferences should be narrowly focused.

This will likely change at later ABCD assessments (e.g. reliability of youth self‐report will increase; see Dorn et al., 2006), and our empirical measure comparison will have to be repeated, with unique considerations required for longitudinal assessments. For example, if data reliability and missingness are problems around pubertal onset, then early maturers will be misrepresented in early ABCD assessments, but *late* maturers will be misrepresented at future assessments. The ABCD Study is poised to provide rich data for future longitudinal multitrait multimethod analyses that may speak to shifts in the (likely sex‐related) predictive utility of youth‐ versus parent‐reports (see Grimm, Pianta, & Konold, 2009). Until then, researchers leveraging these data should prioritize parent‐reports in early adolescence, and beyond that, consider comparing youth and parent PDS reports (e.g. on missingness and reliability) to understand potential impacts of report source on study inferences.

Moreover, physical features are not equal in their consequences. Secondary sex characteristics differ both in social signal and in developmental timetable, likely contributing to some differences detected between the ABCD categorical and PDS continuous measures, which include different items. For example, breast development is considered in both measures, occurs early in puberty, and is visible to others, whereas menarche, which is emphasized in the ABCD categorical measure, has meaning for the adolescent experiencing it, but can be hidden and occurs late in puberty. Both measures are also composite scores, reflecting a youth's overall development but combining features that develop on different timetables and are differently linked to endocrine processes (e.g. adrenal, gonadal) that may be best reflected by other scores derived from the PDS (e.g. Shirtcliff et al., 2009).

Importantly, the systematic review highlighted the problem that papers often do not contain sufficient information about pubertal assessment; this includes whether the ABCD categorical measure was used as well as aspects of measurement shown to differ across the categorical and PDS continuous measures, such as source of report and data missingness (Figure 2). The advancement of puberty science requires that essential details are provided to ensure reproducibility and enable replication (Loken & Gelman, 2017).

Finally, we did not investigate whether the ABCD categorical and PDS continuous measures differentially predicted outcomes because links are unique and complex, requiring careful consideration in focused investigations. For example, pubertal changes may not be linearly related to neural and behavioral changes. Some changes in brain anatomy, such as gray matter volume, are hypothesized to result from hormonal changes occurring in mid‐to‐late puberty (Vijayakumar, de Macks, Shirtcliff, & Pfeifer, 2018). Such effects are unlikely to be captured in linear analyses in a sample of youth representing the range of puberty, but rather require specific analyses reflecting an inflection at mid‐puberty (e.g. polynomial regressions or growth curves) that may be premature to investigate when participants are only age 10 or 11. Furthermore, behavioral changes that result from social reactions to physical development are likely to be tied to changes in specific physical features (not composite scores of overall development), and these associations are likely to be nonlinear (e.g. behavioral changes that result from social responses to breast development early in puberty).

## Conclusions

The impressive size and accessibility of ABCD Study® data are eliciting new interest in the neural and behavioral consequences of adolescent development and in separating effects of age versus puberty. The complexity of pubertal development, however, needs to be addressed with thoughtful conceptualizations and analyses using PDS measures beyond the categorical measure provided in data releases, and often with longitudinal data across the pubertal transition (eschewing the limited hormone data). Ultimately, there is no single best way to describe pubertal development; research questions should drive the data, measures, and models used (Berenbaum et al., 2015; Deardorff et al., 2019; Dorn et al., 2006; Mendle et al., 2019).

## Ethics statement

Parents provided written informed consent and youth provided assent, following each of the Institutional Review Board‐approved protocols at the 21 sites of the ABCD Study. This investigation was approved by the Institutional Review Board of the University of Michigan Medical School (HUM00211215, date of approval: 1/28/2022).

**Key points**

**What's known**

- The Adolescent Brain Cognitive Development^SM^ Study (ABCD Study®) has significant potential to reveal the nature, causes, context, and consequences of pubertal development in diverse American youth.

**What's new**

- Review of studies using ABCD puberty data reveals extensive use of the summary categorical measure provided in data releases. Analyses of ABCD data from self‐ and parent‐report on the Pubertal Development Scale at baseline and Year 1 follow‐up assessments show advantages of an alternative widely used continuous summary measure over the categorical measure in terms of missing data, developmental distributions, and psychometrics.

**What's relevant**

- This work has relevance for studies using ABCD Study® data to understand pubertal influences on adolescent development and to ultimately promote the health and well‐being of youth.

## Supporting information


**Appendix S1.** ABCD categorical measure scoring formula for female youth.
**Appendix S2.** Alternative scoring methods for the PDS.
**Appendix S3.** Studies included in the systematic review.
**Appendix S4.** Sibling exclusion criteria in the empirical comparison.

## Data Availability

The de‐identified data and a data dictionary are already openly available with the completion of a data use agreement through the ABCD Study Release 5.0, NDA Study #2147, DOI: 10.15154/8873‐zj65.
