# Supplementary material for: Research Review: On the (mis)use of puberty data in the ABCD Study® – a systematic review, problem illustration, and path forward
Source: J Child Psychol Psychiatry. 2025 Aug 25;67(1):138–51. doi: 10.1111/jcpp.70035 (PMC12699133; doi:10.1111/jcpp.70035)
Supplement: Supplementary file 1 — Appendix S1. ABCD categorical measure scoring formula for female youth. Appendix S2. Alternative scoring methods for the PDS. Appendix S3. Studies included in the systematic review. Appendix S4. Sibling exclusion criteria in the empirical comparison. [file JCPP-67-138-s001.docx]

**Supplementary Material for**

**“*On the (Mis)Use of Puberty Data in the ABCD Study®:***

***A Systematic Review, Problem Illustration, and Path Forward*”**

**Appendix S1: ABCD categorical measure scoring formula for female youth**

The ABCD categorical score for female adolescents is not a simple sum of responses to PDS items; the presence of menarche is a determinative factor in assigning a score of 4:
1-prepuberty: (body hair+breast development)=2 and menarche=1; 
2-early puberty: (body hair+breast development)=3 and menarche=1; 
3-mid puberty: (body hair+breast development)>3 and menarche=1; 
4-late puberty: (body hair+breast development)<=7 and menarche=4; 
5-post puberty: (body hair+breast development)=8 and menarche=4;

**Appendix S2: Alternative scoring methods for the PDS**

Although the categorical measure is provided in ABCD data releases, other measures derived from the PDS are also discussed in the main papers describing ABCD puberty data (Cheng et al., 2021; Herting et al., 2021), including an average PDS score and two average scores suggested to separately index gonadarche (reflected by items like breast development in female youth and voice changes in male youth) and adrenarche (reflected by items like skin changes; Shirtcliff et al., 2009).

**References**

Cheng, T. W., Magis-Weinberg, L., Guazzelli Williamson, V., Ladouceur, C. D., Whittle, S. L., Herting, M. M., Uban, K. A., Byrne, M. L., Barendse, M. E., & Shirtcliff, E. A. (2021). A researcher’s guide to the measurement and modeling of puberty in the ABCD Study® at baseline. *Frontiers in Endocrinology*, *12*, 608575. <https://doi.org/10.3389/fendo.2021.608575>

Herting, M. M., Uban, K. A., Gonzalez, M. R., Baker, F. C., Kan, E. C., Thompson, W. K., Granger, D. A., Albaugh, M. D., Anokhin, A. P., & Bagot, K. S. (2021). Correspondence between perceived pubertal development and hormone levels in 9-10 year-olds from the adolescent brain cognitive development study. *Frontiers in Endocrinology*, *11*, article 549928. <https://doi.org/10.3389/fendo.2020.549928>

Shirtcliff, E. A., Dahl, R. E., & Pollak, S. D. (2009). Pubertal development: Correspondence between hormonal and physical development. *Child Development*, *80*(2), 327-337. <https://doi.org/10.1111/j.1467-8624.2009.01263.x>

**Appendix S3: Studies included in the systematic review**

Adeli, E., Zhao, Q., Zahr, N. M., Goldstone, A., Pfefferbaum, A., Sullivan, E. V., & Pohl, K. M. (2020). Deep learning identifies morphological determinants of sex differences in the pre-adolescent brain. *NeuroImage*, *223*, 117293. https://doi.org/10.1016/j.neuroimage.2020.117293

Adise, S., Allgaier, N., Laurent, J., Hahn, S., Chaarani, B., Owens, M., Yuan, D., Nyugen, P., Mackey, S., Potter, A., & Garavan, H. P. (2021). Multimodal brain predictors of current weight and weight gain in children enrolled in the ABCD Study ®. *Developmental Cognitive Neuroscience*, *49*, 100948. https://doi.org/10.1016/j.dcn.2021.100948

Adise, S., Boutelle, K. N., Rezvan, P. H., Kan, E., Rhee, K. E., Goran, M. I., & Sowell, E. R. (2024). Sex-specific impulsivity, but not other facets of executive function, predicts fat and sugar intake two-years later amongst adolescents with a healthy weight: Findings from the ABCD study. *Appetite*, *192*, 107081. https://doi.org/10.1016/j.appet.2023.107081

Adise, S., Marshall, A. T., Hahn, S., Zhao, S., Kan, E., Rhee, K. E., Herting, M. M., & Sowell, E. R. (2023). Longitudinal assessment of brain structure and behaviour in youth with rapid weight gain: Potential contributing causes and consequences. *Pediatric Obesity*, *18*(2). https://doi.org/10.1111/ijpo.12985

Adise, S., Marshall, A. T., Kan, E., Gonzalez, M. R., & Sowell, E. R. (2023). Relating neighborhood deprivation to childhood obesity in the ABCD study: Evidence for theories of neuroinflammation and neuronal stress. *Health Psychology*, *42*(12), 868–877. https://doi.org/10.1037/hea0001250

Adise, S., Marshall, A. T., Kan, E., & Sowell, E. R. (2022). Access to quality health resources and environmental toxins affect the relationship between brain structure and BMI in a sample of pre and early adolescents. *Frontiers in Public Health*, *10*. https://doi.org/10.3389/fpubh.2022.1061049

Adise, S., Ottino‐Gonzalez, J., Goedde, L., Marshall, A. T., Kan, E., Rhee, K. E., Goran, M. I., & Sowell, E. R. (2023). Variation in executive function relates to BMI increases in youth who were initially of a healthy weight in the ABCD Study. *Obesity*, *31*(11), 2809–2821. https://doi.org/10.1002/oby.23811

Adise, S., Ottino‐Gonzalez, J., Hayati Rezvan, P., Kan, E., Rhee, K. E., Goran, M. I., & Sowell, E. R. (2024). Smaller subcortical volume relates to greater weight gain in girls with initially healthy weight. *Obesity*, *32*(7), 1389–1400. https://doi.org/10.1002/oby.24028

Adise, S., Palmer, C. E., Sheth, C., Marshall, A. T., Baker, F. C., Brown, S. A., Chang, L., Clark, D. B., Dagher, R. K., Diaz, V., Haist, F., Herting, M. M., Huber, R. S., LeBlanc, K., Lee, K. C., Liang, H., Linkersdörfer, J., Lisdahl, K. M., Ma, J., … Sowell, E. R. (2024). Associations between perinatal risk and physical health in pre-adolescence in the Adolescent Brain Cognitive Development (ABCD) Study®: The unexpected relationship with sleep disruption. *Pediatric Research*, *96*(7), 1834–1842. https://doi.org/10.1038/s41390-024-03288-z

Allen, B., Lane, M., Steeves, E. A., & Raynor, H. (2022). Using explainable artificial intelligence to discover interactions in an ecological model for obesity. *International Journal of Environmental Research and Public Health*, *19*(15), 9447. https://doi.org/10.3390/ijerph19159447

Argabright, S. T., Moore, T. M., Visoki, E., DiDomenico, G. E., Taylor, J. H., & Barzilay, R. (2022). Association between racial/ethnic discrimination and pubertal development in early adolescence. *Psychoneuroendocrinology*, *140*, 105727. https://doi.org/10.1016/j.psyneuen.2022.105727

Assari, S., Najand, B., Najand, I., & Grace, S. (2024). Behavioral and psychosocial correlates of hope among youth. *Journal of Medicine, Surgery, and Public Health*, *2*, 100088. https://doi.org/10.1016/j.glmedi.2024.100088

Assari, S., Najand, B., & Sheikhattari, P. (2024). Association between nucleus accumbens volume and future tobacco and marijuana use in early adolescence. *Journal of Medicine, Surgery, and Public Health*, *2*, 100071. https://doi.org/10.1016/j.glmedi.2024.100071

Assari, S., & Sheikhattari, P. (2024). Sex differences in the relationship between nucleus accumbens volume and youth tobacco or marijuana use following stressful life events. *Journal of Mental Health & Clinical Psychology*, *8*(2), 1–13. https://doi.org/10.29245/2578-2959/2024/2.1305

Assari, S., & Zare, H. (2024). Extreme heat exposure is associated with higher socioeconomic disadvantage and elevated youth delinquency. *Trends Journal of Sciences Research*, *3*(1), 15–28. https://doi.org/10.31586/jsmhes.2024.1044

Baboli, R., Cao, M., Halperin, J. M., & Li, X. (2022). Distinct thalamic and frontal neuroanatomical substrates in children with familial vs. non-familial Attention-Deficit/Hyperactivity Disorder (ADHD). *Brain Sciences*, *13*(1), 46. https://doi.org/10.3390/brainsci13010046

Baranger, D. A. A., Miller, A. P., Gorelik, A. J., Paul, S. E., Hatoum, A. S., Johnson, E. C., Colbert, S. M. C., Smyser, C. D., Rogers, C. E., Bijsterbosch, J. D., Agrawal, A., & Bogdan, R. (2024). Prenatal cannabis exposure, the brain, and psychopathology during early adolescence. *Nature Mental Health*, *2*(8), 975–986. https://doi.org/10.1038/s44220-024-00281-7

Baranger, D. A. A., Paul, S. E., Colbert, S. M. C., Karcher, N. R., Johnson, E. C., Hatoum, A. S., & Bogdan, R. (2022). Association of mental health burden with prenatal cannabis exposure from childhood to early adolescence. *JAMA Pediatrics*, *176*(12), 1261. https://doi.org/10.1001/jamapediatrics.2022.3191

Barendse, M. E. A., Swartz, J. R., Taylor, S. L., Fine, J. R., Shirtcliff, E. A., Yoon, L., McMillan, S. J., Tully, L. M., & Guyer, A. E. (2024). Sex and pubertal variation in reward-related behavior and neural activation in early adolescents. *Developmental Cognitive Neuroscience*, *66*, 101358. https://doi.org/10.1016/j.dcn.2024.101358

Barton, A. W., Yu, T., Gong, Q., Chen, E., Miller, G. E., & Brody, G. H. (2024). Skin-deep resilience and early adolescence: Neighborhood disadvantage, executive functioning, and pubertal development in minority youth. *Journal of Youth and Adolescence*, *53*(2), 284–293. https://doi.org/10.1007/s10964-023-01911-6

Beck, D., Ferschmann, L., MacSweeney, N., Norbom, L. B., Wiker, T., Aksnes, E., Karl, V., Dégeilh, F., Holm, M., Mills, K. L., Andreassen, O. A., Agartz, I., Westlye, L. T., von Soest, T., & Tamnes, C. K. (2023). Puberty differentially predicts brain maturation in male and female youth: A longitudinal ABCD Study. *Developmental Cognitive Neuroscience*, *61*, 101261. https://doi.org/10.1016/j.dcn.2023.101261

Ben-Asher, E., Porter, B. M., & Church, J. A. (2024). Distinct constellations of common risk factors differentially relate to executive-function ability in children. *Psychological Science*, *35*(5), 489–503. https://doi.org/10.1177/09567976241235931

Bernanke, J., Luna, A., Chang, L., Bruno, E., Dworkin, J., & Posner, J. (2022). Structural brain measures among children with and without ADHD in the Adolescent Brain and Cognitive Development Study cohort: A cross-sectional US population-based study. *The Lancet Psychiatry*, *9*(3), 222–231. https://doi.org/10.1016/S2215-0366(21)00505-8

Betts, S. S., Adise, S., Hayati Rezvan, P., Marshall, A. T., Kan, E., Johnson, D. L., & Sowell, E. R. (2023). Socioeconomic adversity and weight gain during the COVID-19 pandemic. *JAMA Pediatrics*, *177*(10), 1102. https://doi.org/10.1001/jamapediatrics.2023.2823

Botdorf, M., Dunstan, J., Sorcher, L., Dougherty, L. R., & Riggins, T. (2022). Socioeconomic disadvantage and episodic memory ability in the ABCD sample: Contributions of hippocampal subregion and subfield volumes. *Developmental Cognitive Neuroscience*, *57*, 101138. https://doi.org/10.1016/j.dcn.2022.101138

Bottenhorn, K. L., Cardenas-Iniguez, C., Mills, K. L., Laird, A. R., & Herting, M. M. (2023). Profiling intra- and inter-individual differences in brain development across early adolescence. *NeuroImage*, *279*, 120287. https://doi.org/10.1016/j.neuroimage.2023.120287

Brooks, S. J., Jones, V. O., Wang, H., Deng, C., Golding, S. G. H., Lim, J., Gao, J., Daoutidis, P., & Stamoulis, C. (2024). Community detection in the human connectome: Method types, differences and their impact on inference. *Human Brain Mapping*, *45*(5). https://doi.org/10.1002/hbm.26669

Brooks, S. J., Katz, E. S., & Stamoulis, C. (2022). Shorter duration and lower quality sleep have widespread detrimental effects on developing functional brain networks in early adolescence. *Cerebral Cortex Communications*, *3*(1). https://doi.org/10.1093/texcom/tgab062

Brooks, S. J., Parks, S. M., & Stamoulis, C. (2021). Widespread positive direct and indirect effects of regular physical activity on the developing functional connectome in early adolescence. *Cerebral Cortex*, *31*(10), 4840–4852. https://doi.org/10.1093/cercor/bhab126

Cao, Z., Cupertino, R. B., Ottino-Gonzalez, J., Murphy, A., Pancholi, D., Juliano, A., Chaarani, B., Albaugh, M., Yuan, D., Schwab, N., Stafford, J., Goudriaan, A. E., Hutchison, K., Li, C.-S. R., Luijten, M., Groefsema, M., Momenan, R., Schmaal, L., Sinha, R., … Garavan, H. (2023). Cortical profiles of numerous psychiatric disorders and normal development share a common pattern. *Molecular Psychiatry*, *28*(2), 698–709. https://doi.org/10.1038/s41380-022-01855-6

Chaarani, B., Hahn, S., Allgaier, N., Adise, S., Owens, M. M., Juliano, A. C., Yuan, D. K., Loso, H., Ivanciu, A., Albaugh, M. D., Dumas, J., Mackey, S., Laurent, J., Ivanova, M., Hagler, D. J., Cornejo, M. D., Hatton, S., Agrawal, A., Aguinaldo, L., … Garavan, H. P. (2021). Baseline brain function in the preadolescents of the ABCD Study. *Nature Neuroscience*, *24*(8), 1176–1186. https://doi.org/10.1038/s41593-021-00867-9

Chaarani, Bader, Ortigara, J., Yuan, D., Loso, H., Potter, A., & Garavan, H. P. (2022). Association of video gaming with cognitive performance among children. *JAMA Network Open*, *5*(10), e2235721. https://doi.org/10.1001/jamanetworkopen.2022.35721

Chaku, N., & Barry, K. (2024). Exploring profiles of hormone exposure: Associations with cognition in a population‐based cohort of early adolescents. *Infant and Child Development*, *33*(2). https://doi.org/10.1002/icd.2415

Chaku, N., Barry, K., Fowle, J., & Hoyt, L. T. (2022). Understanding patterns of heterogeneity in executive functioning during adolescence: Evidence from population‐level data. *Developmental Science*, *25*(6). https://doi.org/10.1111/desc.13256

Cheng, W., Rolls, E., Gong, W., Du, J., Zhang, J., Zhang, X.-Y., Li, F., & Feng, J. (2021). Sleep duration, brain structure, and psychiatric and cognitive problems in children. *Molecular Psychiatry*, *26*(8), 3992–4003. https://doi.org/10.1038/s41380-020-0663-2

Conley, M. I., Skalaban, L. J., Rapuano, K. M., Gonzalez, R., Laird, A. R., Dick, A. S., Sutherland, M. T., Watts, R., & Casey, B. J. (2021). Altered hippocampal microstructure and function in children who experienced Hurricane Irma. *Developmental Psychobiology*, *63*(5), 864–877. https://doi.org/10.1002/dev.22071

Cooper, R., Di Biase, M. A., Bei, B., Quach, J., & Cropley, V. (2023). Associations of changes in sleep and emotional and behavioral problems from late childhood to early adolescence. *JAMA Psychiatry*, *80*(6), 585. https://doi.org/10.1001/jamapsychiatry.2023.0379

Dai, H. D., Doucet, G. E., Wang, Y., Puga, T., Samson, K., Xiao, P., & Khan, A. S. (2022). Longitudinal assessments of neurocognitive performance and brain structure associated with initiation of tobacco use in children, 2016 to 2021. *JAMA Network Open*, *5*(8), e2225991. https://doi.org/10.1001/jamanetworkopen.2022.25991

de Lacy, N., & Ramshaw, M. J. (2023). Selectively predicting the onset of ADHD, oppositional defiant disorder, and conduct disorder in early adolescence with high accuracy. *Frontiers in Psychiatry*, *14*. https://doi.org/10.3389/fpsyt.2023.1280326

Dehestani, N., Whittle, S., Vijayakumar, N., & Silk, T. J. (2023). Developmental brain changes during puberty and associations with mental health problems. *Developmental Cognitive Neuroscience*, *60*, 101227. https://doi.org/10.1016/j.dcn.2023.101227

Del Toro, J., Anderson, R. E., Sun, X., & Lee, R. M. (2024). Early adolescents’ ethnic–racial discrimination and pubertal development: Parents’ ethnic–racial identities promote adolescents’ resilience. *American Psychologist*, *79*(8), 1109–1122. https://doi.org/10.1037/amp0001284

Demidenko, M. I., Ip, K. I., Kelly, D. P., Constante, K., Goetschius, L. G., & Keating, D. P. (2021). Ecological stress, amygdala reactivity, and internalizing symptoms in preadolescence: Is parenting a buffer? *Cortex*, *140*, 128–144. https://doi.org/10.1016/j.cortex.2021.02.032

Demidenko, M. I., Kelly, D. P., Hardi, F. A., Ip, K. I., Lee, S., Becker, H., Hong, S., Thijssen, S., Luciana, M., & Keating, D. P. (2022). Mediating effect of pubertal stages on the family environment and neurodevelopment: An open-data replication and multiverse analysis of an ABCD Study®. *Neuroimage: Reports*, *2*(4), 100133. https://doi.org/10.1016/j.ynirp.2022.100133

DeVille, D. C., Whalen, D., Breslin, F. J., Morris, A. S., Khalsa, S. S., Paulus, M. P., & Barch, D. M. (2020). Prevalence and family-related factors associated with suicidal ideation, suicide attempts, and self-injury in children aged 9 to 10 years. *JAMA Network Open*, *3*(2), e1920956. https://doi.org/10.1001/jamanetworkopen.2019.20956

Dube, S., Ivanova, M., & Potter, A. (2021). “I don’t understand”: Who is missed when we ask early adolescents, “Are you transgender”? *Archives of Sexual Behavior*, *50*(3), 741–745. https://doi.org/10.1007/s10508-021-01986-x

Elton, A., Lewis, B., & Nixon, S. J. (2024). The effects of adverse life events on brain development in the ABCD study®: A propensity-weighted analysis. *Molecular Psychiatry*. https://doi.org/10.1038/s41380-024-02850-9

Ertel, K. A., Okuzono, S. S., Beyer, L. N., Pintro, K., Cuevas, A. G., & Slopen, N. (2024). Neighborhood opportunity and obesity in early adolescence: Differential associations by sex. *Journal of Adolescent Health*, *75*(2), 314–322. https://doi.org/10.1016/j.jadohealth.2024.04.009

Fan, H., Liu, Z., Wu, X., Yu, G., Gu, X., Kuang, N., Zhang, K., Liu, Y., Jia, T., Sahakian, B. J., Robbins, T. W., Schumann, G., Cheng, W., Feng, J., Becker, B., & Zhang, J. (2023). Decoding anxiety–impulsivity subtypes in preadolescent internalising disorders: Findings from the Adolescent Brain Cognitive Development study. *The British Journal of Psychiatry*, *223*(6), 542–554. https://doi.org/10.1192/bjp.2023.107

Freeman, C., Olino, T., Barbeau, E. B., Weinberg, A., & Chai, X. (2023). Family history of depression and neural reward sensitivity: Findings from the Adolescent Brain Cognitive Development Study. *Biological Psychiatry: Cognitive Neuroscience and Neuroimaging*, *8*(6), 620–629. https://doi.org/10.1016/j.bpsc.2022.09.015

Fung, H., Yeo, B. T. T., Chen, C., Lo, J. C., Chee, M. W. L., & Ong, J. L. (2023). Adherence to 24-hour movement recommendations and health indicators in early adolescence: Cross-sectional and longitudinal associations in the Adolescent Brain Cognitive Development Study. *Journal of Adolescent Health*, *72*(3), 460–470. https://doi.org/10.1016/j.jadohealth.2022.10.019

Gadassi Polack, R., Mollick, J. A., Keren, H., Joormann, J., & Watts, R. (2023). Neural responses to reward valence and magnitude from pre- to early adolescence. *NeuroImage*, *275*, 120166. https://doi.org/10.1016/j.neuroimage.2023.120166

Gard, A. M., Hyde, L. W., Heeringa, S. G., West, B. T., & Mitchell, C. (2023). Why weight? Analytic approaches for large-scale population neuroscience data. *Developmental Cognitive Neuroscience*, *59*, 101196. https://doi.org/10.1016/j.dcn.2023.101196

George, G. C., Heyn, S. A., Russell, J. D., Keding, T. J., & Herringa, R. J. (2024). Parent psychopathology and behavioral effects on child brain–symptom networks in the ABCD study. *Journal of the American Academy of Child & Adolescent Psychiatry*, *63*(10), 1024–1034. https://doi.org/10.1016/j.jaac.2023.12.016

Goldstone, A., Javitz, H. S., Claudatos, S. A., Buysse, D. J., Hasler, B. P., de Zambotti, M., Clark, D. B., Franzen, P. L., Prouty, D. E., Colrain, I. M., & Baker, F. C. (2020). Sleep disturbance predicts depression symptoms in early adolescence: Initial findings from the Adolescent Brain Cognitive Development Study. *Journal of Adolescent Health*, *66*(5), 567–574. https://doi.org/10.1016/j.jadohealth.2019.12.005

Gonçalves, P. D., Martins, S. S., Gebru, N. M., Ryan-Pettes, S. R., Allgaier, N., Potter, A., Thompson, W. K., Johnson, M. E., Garavan, H., Talati, A., & Albaugh, M. D. (2024). Associations between family history of alcohol and/or substance use problems and frontal cortical development from 9 to 13 years of age: A longitudinal analysis of the ABCD study. *Biological Psychiatry Global Open Science*, *4*(2), 100284. https://doi.org/10.1016/j.bpsgos.2023.100284

Gong, W., Rolls, E. T., Du, J., Feng, J., & Cheng, W. (2021). Brain structure is linked to the association between family environment and behavioral problems in children in the ABCD Study. *Nature Communications*, *12*(1), 3769. https://doi.org/10.1038/s41467-021-23994-0

Gorelik, A. J., Paul, S. E., Karcher, N. R., Johnson, E. C., Nagella, I., Blaydon, L., Modi, H., Hansen, I. S., Colbert, S. M. C., Baranger, D. A. A., Norton, S. A., Spears, I., Gordon, B., Zhang, W., Hill, P. L., Oltmanns, T. F., Bijsterbosch, J. D., Agrawal, A., Hatoum, A. S., & Bogdan, R. (2023). A phenome-wide association study (PheWAS) of late onset alzheimer disease genetic risk in children of European ancestry at middle childhood: Results from the ABCD Study. *Behavior Genetics*, *53*(3), 249–264. https://doi.org/10.1007/s10519-023-10140-3

Green, R., Wolf, B. J., Chen, A., Kirkland, A. E., Ferguson, P. L., Browning, B. D., Bryant, B. E., Tomko, R. L., Gray, K. M., Mewton, L., & Squeglia, L. M. (2024). Predictors of substance use initiation by early adolescence. *American Journal of Psychiatry*, *181*(5), 423–433. https://doi.org/10.1176/appi.ajp.20230882

Guberman, G. I., Stojanovski, S., Nishat, E., Ptito, A., Bzdok, D., Wheeler, A. L., & Descoteaux, M. (2022). Multi-tract multi-symptom relationships in pediatric concussion. *ELife*, *11*. https://doi.org/10.7554/eLife.70450

Guberman, G. I., Theaud, G., Hawes, S. W., Ptito, A., Descoteaux, M., & Hodgins, S. (2024). White matter microstructure, traumatic brain injury, and disruptive behavior disorders in girls and boys. *Frontiers in Neuroscience*, *18*. https://doi.org/10.3389/fnins.2024.1391407

Gunther, K. E., Petrie, D., Pérez-Edgar, K., & Geier, C. (2023). Relations between executive functioning and internalizing symptoms vary as a function of frontoparietal-amygdala resting state connectivity. *Research on Child and Adolescent Psychopathology*, *51*(6), 775–788. https://doi.org/10.1007/s10802-023-01025-4

Hall, P. A., Best, J. R., Beaton, E. A., Sakib, M. N., & Danckert, J. (2023). Morphology of the prefrontal cortex predicts body composition in early adolescence: Cognitive mediators and environmental moderators in the ABCD Study. *Social Cognitive and Affective Neuroscience*, *18*(1). https://doi.org/10.1093/scan/nsab104

Harju-Seppänen, J., Irizar, H., Bramon, E., Blakemore, S.-J., Mason, L., & Bell, V. (2022). Reward processing in children with psychotic-like experiences. *Schizophrenia Bulletin Open*, *3*(1). https://doi.org/10.1093/schizbullopen/sgab054

Hehr, A., Huntley, E. D., & Marusak, H. A. (2023). Getting a good night’s sleep: Associations between sleep duration and parent-reported sleep quality on default mode network connectivity in youth. *Journal of Adolescent Health*, *72*(6), 933–942. https://doi.org/10.1016/j.jadohealth.2023.01.010

Herting, M. M., Uban, K. A., Gonzalez, M. R., Baker, F. C., Kan, E. C., Thompson, W. K., Granger, D. A., Albaugh, M. D., Anokhin, A. P., Bagot, K. S., Banich, M. T., Barch, D. M., Baskin-Sommers, A., Breslin, F. J., Casey, B. J., Chaarani, B., Chang, L., Clark, D. B., Cloak, C. C., … Sowell, E. R. (2021). Correspondence between perceived pubertal development and hormone levels in 9-10 year-olds from the Adolescent Brain Cognitive Development Study. *Frontiers in Endocrinology*, *11*(February), 1–22. https://doi.org/10.3389/fendo.2020.549928

Holm, M. C., Leonardsen, E. H., Beck, D., Dahl, A., Kjelkenes, R., de Lange, A.-M. G., & Westlye, L. T. (2023). Linking brain maturation and puberty during early adolescence using longitudinal brain age prediction in the ABCD cohort. *Developmental Cognitive Neuroscience*, *60*, 101220. https://doi.org/10.1016/j.dcn.2023.101220

Holt-Gosselin, B., Keding, T. J., Rodrigues, K., Rueter, A., Hendrickson, T. J., Perrone, A., Byington, N., Houghton, A., Miranda-Dominguez, O., Feczko, E., Fair, D. A., Joormann, J., & Gee, D. G. (2024). Familial risk for depression moderates neural circuitry in healthy preadolescents to predict adolescent depression symptoms in the Adolescent Brain Cognitive Development (ABCD) Study. *Developmental Cognitive Neuroscience*, *68*, 101400. https://doi.org/10.1016/j.dcn.2024.101400

Hull, S., Origlio, J., Noyola, N., Henin, A., & Liu, R. T. (2025). Dimensions of experienced gender and prospective self-injurious thoughts and behaviors in preadolescent children: A national study. *Journal of Affective Disorders*, *369*, 467–474. https://doi.org/10.1016/j.jad.2024.10.033

Ji, W., Li, G., Hu, Y., Zhang, W., Wang, J., Jiang, F., Zhang, Y., Wu, F., Wei, X., Li, Y., Gao, X., Manza, P., Volkow, N. D., Wang, G.-J., & Zhang, Y. (2024). Associations among birth weight, adrenarche, brain morphometry, and cognitive function in preterm children ages 9 to 11 years. *Biological Psychiatry: Cognitive Neuroscience and Neuroimaging*, *9*(9), 871–881. https://doi.org/10.1016/j.bpsc.2024.02.012

Johnson, E. I., Planalp, E. M., Williams, D. T., & Poehlmann, J. (2024). Parental incarceration and health risks in a population-based study of U.S. early adolescents: Results among racialized groups. *SSM - Population Health*, *27*, 101702. https://doi.org/10.1016/j.ssmph.2024.101702

Kaltenhauser, S., Weber, C. F., Lin, H., Mozayan, A., Malhotra, A., Constable, R. T., Acosta, J. N., Falcone, G. J., Taylor, S. N., Ment, L. R., Sheth, K. N., & Payabvash, S. (2023). Association of body mass index and waist circumference with imaging metrics of brain integrity and functional connectivity in children aged 9 to 10 years in the US, 2016-2018. *JAMA Network Open*, *6*(5), e2314193. https://doi.org/10.1001/jamanetworkopen.2023.14193

Kaplan, C. M., Schrepf, A., Boehnke, K. F., He, Y., Smith, T., Williams, D. A., Bergmans, R., Voepel-Lewis, T., Hassett, A. L., Harris, R. E., Clauw, D. J., Beltz, A. M., & Harte, S. E. (2023). Risk factors for the development of multisite pain in children. *The Clinical Journal of Pain*. https://doi.org/10.1097/AJP.0000000000001148

Karcher, N. R., O’Hare, K., Jay, S. Y., & Grattan, R. (2023). Strengthening associations between psychotic like experiences and suicidal ideation and behavior across middle childhood and early adolescence. *Psychological Medicine*, *53*(13), 6002–6010. https://doi.org/10.1017/S0033291722003166

Kennedy, J. T., Harms, M. P., Korucuoglu, O., Astafiev, S. V., Barch, D. M., Thompson, W. K., Bjork, J. M., & Anokhin, A. P. (2022). Reliability and stability challenges in ABCD task fMRI data. *NeuroImage*, *252*, 119046. https://doi.org/10.1016/j.neuroimage.2022.119046

Kraft, D., Alnæs, D., & Kaufmann, T. (2023). Domain adapted brain network fusion captures variance related to pubertal brain development and mental health. *Nature Communications*, *14*(1), 6698. https://doi.org/10.1038/s41467-023-41839-w

Ku, B. S., Yuan, Q., Arias-Magnasco, A., Lin, B. D., Walker, E. F., Druss, B. G., Ren, J., van Os, J., & Guloksuz, S. (2024). Associations between genetic risk, physical activities, and distressing psychotic-like experiences. *Schizophrenia Bulletin*. https://doi.org/10.1093/schbul/sbae141

Kuang, N., Liu, Z., Yu, G., Wu, X., Becker, B., Fan, H., Peng, S., Zhang, K., Zhao, J., Kang, J., Dong, G., Zhao, X., Sahakian, B. J., Robbins, T. W., Cheng, W., Feng, J., Schumann, G., Palaniyappan, L., & Zhang, J. (2023). Neurodevelopmental risk and adaptation as a model for comorbidity among internalizing and externalizing disorders: Genomics and cell-specific expression enriched morphometric study. *BMC Medicine*, *21*(1), 291. https://doi.org/10.1186/s12916-023-02920-9

Kulisch, L. K., Arumäe, K., Briley, D. A., & Vainik, U. (2023). Triangulating causality between childhood obesity and neurobehavior: Behavioral genetic and longitudinal evidence. *Developmental Science*, *26*(6). https://doi.org/10.1111/desc.13392

Kunitoki, K., Hughes, D., Elyounssi, S., Hopkinson, C. E., Bazer, O. M., Eryilmaz, H., Dunn, E. C., Lee, P. H., Doyle, A. E., & Roffman, J. L. (2023). Youth team sports participation associates with reduced dimensional psychopathology through interaction with biological risk factors. *Biological Psychiatry Global Open Science*, *3*(4), 875–883. https://doi.org/10.1016/j.bpsgos.2023.02.001

Laurent, J. S., Watts, R., Adise, S., Allgaier, N., Chaarani, B., Garavan, H., Potter, A., & Mackey, S. (2020). Associations among body mass index, cortical thickness, and executive function in children. *JAMA Pediatrics*, *174*(2), 170. https://doi.org/10.1001/jamapediatrics.2019.4708

Lawrence, K. E., Abaryan, Z., Laltoo, E., Hernandez, L. M., Gandal, M. J., McCracken, J. T., & Thompson, P. M. (2023). White matter microstructure shows sex differences in late childhood: Evidence from 6797 children. *Human Brain Mapping*, *44*(2), 535–548. https://doi.org/10.1002/hbm.26079

Lewis-de los Angeles, W. W., & Liu, R. T. (2021). History of depression, elevated body mass index, and waist-to-height ratio in preadolescent children. *Psychosomatic Medicine*, *83*(9), 1075–1081. https://doi.org/10.1097/PSY.0000000000000982

Li, R., Groenewald, C., Tham, S. W., Rabbitts, J. A., Ward, T. M., & Palermo, T. M. (2024). Influence of chronotype on pain incidence during early adolescence. *Pain*, *165*(11), 2595–2605. https://doi.org/10.1097/j.pain.0000000000003271

Li, R., Lopez, D. A., & Palermo, T. M. (2023). Pubertal development and pain incidence and characteristics in children: A 1-year prospective cohort study of a national sample. *The Journal of Pain*, *24*(4), 89. https://doi.org/10.1016/j.jpain.2023.02.255

Li, X., Motwani, C., Cao, M., Martin, E., & Halperin, J. M. (2023). Working memory-related neurofunctional correlates associated with the frontal lobe in children with familial vs. non-familial attention deficit/hyperactivity disorder. *Brain Sciences*, *13*(10), 1469. https://doi.org/10.3390/brainsci13101469

Li, Z. A., Cai, Y., Taylor, R. L., Eisenstein, S. A., Barch, D. M., Marek, S., & Hershey, T. (2023). Associations between socioeconomic status, obesity, cognition, and white matter microstructure in children. *JAMA Network Open*, *6*(6), e2320276. https://doi.org/10.1001/jamanetworkopen.2023.20276

Li, Z. A., Ray, M. K., Gu, Y., Barch, D. M., & Hershey, T. (2024). Weight indices, cognition, and mental health from childhood to early adolescence. *JAMA Pediatrics*, *178*(8), 830. https://doi.org/10.1001/jamapediatrics.2024.1379

Li, Z. A., Samara, A., Ray, M. K., Rutlin, J., Raji, C. A., Shimony, J. S., Sun, P., Song, S.-K., Hershey, T., & Eisenstein, S. A. (2023). Childhood obesity is linked to putative neuroinflammation in brain white matter, hypothalamus, and striatum. *Cerebral Cortex Communications*, *4*(2). https://doi.org/10.1093/texcom/tgad007

Loso, H., Chaarani, B., Dube, S. L., Albaugh, M. D., Cheaito, A., Garavan, H., & Potter, A. (2023). Gender diversity associated with patterns of brain activation seen in populations that experience childhood stress. *Frontiers in Integrative Neuroscience*, *17*. https://doi.org/10.3389/fnint.2023.1084748

Loso, Hannah M., Locke Dube, S., Chaarani, B., Ivanova, M., Garavan, H., Johns, M. M., & Potter, A. S. (2023). Associations between gender nonconformity, school environments, family conflict, and emotional and behavioral health among children ages 10–11. *Journal of Adolescent Health*, *72*(6), 869–876. https://doi.org/10.1016/j.jadohealth.2023.02.008

Loso, Hannah Marie, Dube, S. L., Chaarani, B., Garavan, H., Albaugh, M., Ivanova, M., & Potter, A. (2021). Sex differences in psychopathology in a large cohort of nine and ten-year-olds. *Psychiatry Research*, *302*, 114026. https://doi.org/10.1016/j.psychres.2021.114026

Lowe, C. J., & Bodell, L. P. (2024). Examining neural responses to anticipating or receiving monetary rewards and the development of binge eating in youth. A registered report using data from the Adolescent Brain Cognitive Development (ABCD) study. *Developmental Cognitive Neuroscience*, *67*, 101377. https://doi.org/10.1016/j.dcn.2024.101377

Ludyga, S., & Ishihara, T. (2022). Brain structural changes and the development of interference control in children with ADHD: The predictive value of physical activity and body mass index. *NeuroImage: Clinical*, *35*, 103141. https://doi.org/10.1016/j.nicl.2022.103141

Lunsford‐Avery, J. R., Damme, K. S. F., Vargas, T., Sweitzer, M. M., & Mittal, V. A. (2021). Psychotic‐like experiences associated with sleep disturbance and brain volumes in youth: Findings from the Adolescent Brain Cognitive Development Study. *JCPP Advances*, *1*(4). https://doi.org/10.1002/jcv2.12055

Luo, S., Hsu, E., Lawrence, K. E., Adise, S., Pickering, T. A., Herting, M. M., Buchanan, T., Page, K. A., & Thompson, P. M. (2023). Associations among prenatal exposure to gestational diabetes mellitus, brain structure, and child adiposity markers. *Obesity*, *31*(11), 2699–2708. https://doi.org/10.1002/oby.23901

Ma, J., McGlade, E. C., Huber, R. S., Lyoo, I. K., Renshaw, P. F., & Yurgelun-Todd, D. A. (2023). Overweight/Obesity-related microstructural alterations of the fimbria-fornix in the ABCD study: The role of aerobic physical activity. *PLOS ONE*, *18*(7), e0287682. https://doi.org/10.1371/journal.pone.0287682

Ma, Q., Cui, Y., Han, X., Xiong, Y., Xu, J., Zhao, H., Li, X., Cheng, W., & Zhou, Q. (2024). Association of maternal hypertension during pregnancy with brain structure and behavioral problems in early adolescence. *European Child & Adolescent Psychiatry*, *33*(7), 2173–2187. https://doi.org/10.1007/s00787-023-02305-6

Ma, Q., Wang, H., Rolls, E. T., Xiang, S., Li, J., Li, Y., Zhou, Q., Cheng, W., & Li, F. (2022). Lower gestational age is associated with lower cortical volume and cognitive and educational performance in adolescence. *BMC Medicine*, *20*(1), 424. https://doi.org/10.1186/s12916-022-02627-3

MacSweeney, N., Allardyce, J., Edmondson-Stait, A., Shen, X., Casey, H., Chan, S. W. Y., Cullen, B., Reynolds, R. M., Frangou, S., Kwong, A. S. F., Lawrie, S. M., Romaniuk, L., & Whalley, H. C. (2023). The role of brain structure in the association between pubertal timing and depression risk in an early adolescent sample (the ABCD Study®): A registered report. *Developmental Cognitive Neuroscience*, *60*, 101223. https://doi.org/10.1016/j.dcn.2023.101223

Martinez Agulleiro, L., Castellanos, F. X., Janssen, A., & Baroni, A. (2024). Family discordance in gender identification is not associated with increased depression and anxiety among trans youth. *LGBT Health*, *11*(3), 193–201. https://doi.org/10.1089/lgbt.2023.0143

Martinez, M., Cai, T., Yang, B., Zhou, Z., Shankman, S. A., Mittal, V. A., Haase, C. M., & Qu, Y. (2024). Depressive symptoms during the transition to adolescence: Left hippocampal volume as a marker of social context sensitivity. *Proceedings of the National Academy of Sciences*, *121*(37). https://doi.org/10.1073/pnas.2321965121

Martínez, M., Damme, K. S., Vargas, T., Yang, B., Rompilla, D. J., Stephens, J., Qu, Y., Mittal, V. A., & Haase, C. M. (2024). Longitudinal study of peer victimization, social support, and mental health during early adolescence. *Psychological Medicine*, *54*(9), 1940–1955. https://doi.org/10.1017/S0033291724000035

Mattoni, M., Hopman, H. J., Dadematthews, A., Chan, S. S. M., & Olino, T. M. (2023). Specificity of associations between parental psychopathology and offspring brain structure. *Psychiatry Research: Neuroimaging*, *334*, 111684. https://doi.org/10.1016/j.pscychresns.2023.111684

McKay, C. C., De Jesus, A. V., Peterson, O., Leibenluft, E., & Kircanski, K. (2024). Cross-sectional and longitudinal relations among irritability, attention-deficit/hyperactivity disorder symptoms, and inhibitory control. *Journal of the American Academy of Child & Adolescent Psychiatry*, *63*(10), 1014–1023. https://doi.org/10.1016/j.jaac.2023.10.015

McNeilly, E. A., Saragosa-Harris, N. M., Mills, K. L., Dahl, R. E., & Magis-Weinberg, L. (2022). Reward sensitivity and internalizing symptoms during the transition to puberty: An examination of 9-and 10-year-olds in the ABCD Study. *Developmental Cognitive Neuroscience*, *58*, 101172. https://doi.org/10.1016/j.dcn.2022.101172

Miller, A. P., Baranger, D. A. A., Paul, S. E., Garavan, H., Mackey, S., Tapert, S. F., LeBlanc, K. H., Agrawal, A., & Bogdan, R. (2024). Neuroanatomical variability and substance use initiation in late childhood and early adolescence. *JAMA Network Open*, *7*(12), e2452027. https://doi.org/10.1001/jamanetworkopen.2024.52027

Modabbernia, A., Janiri, D., Doucet, G. E., Reichenberg, A., & Frangou, S. (2021). Multivariate patterns of brain-behavior-environment associations in the Adolescent Brain and Cognitive Development Study. *Biological Psychiatry*, *89*(5), 510–520. https://doi.org/10.1016/j.biopsych.2020.08.014

Modabbernia, A., Michelini, G., Reichenberg, A., Kotov, R., Barch, D., & Frangou, S. (2022). Neural signatures of data-driven psychopathology dimensions at the transition to adolescence. *European Psychiatry*, *65*(1), e12. https://doi.org/10.1192/j.eurpsy.2021.2262

Moore, T. M., Visoki, E., Argabright, S. T., Didomenico, G. E., Sotelo, I., Wortzel, J. D., Naeem, A., Gur, R. C., Gur, R. E., Warrier, V., Guloksuz, S., & Barzilay, R. (2022). Modeling environment through a general exposome factor in two independent adolescent cohorts. *Exposome*, *2*(1). https://doi.org/10.1093/exposome/osac010

Murray, S. B., Alba, C., Duval, C. J., Nagata, J. M., Cabeen, R. P., Lee, D. J., Toga, A. W., Siegel, S. J., & Jann, K. (2023). Aberrant functional connectivity between reward and inhibitory control networks in pre-adolescent binge eating disorder. *Psychological Medicine*, *53*(9), 3869–3878. https://doi.org/10.1017/S0033291722000514

Murray, S. B., Alba, C., Duval, C. J., Nagata, J. M., Ganson, K. T., & Jann, K. (2023). Sex differences in functional connectivity from reward-based regions in pre-adolescent binge eating disorder. *Psychiatry Research*, *324*, 115186. https://doi.org/10.1016/j.psychres.2023.115186

Murray, S. B., Blashill, A. J., & Calzo, J. P. (2022). Prevalence of disordered eating and associations with sex, pubertal maturation, and weight in children in the US. *JAMA Pediatrics*, *176*(10), 1039. https://doi.org/10.1001/jamapediatrics.2022.2490

Murray, S. B., Diaz-Fong, J. P., Duval, C. J., Balkchyan, A. A., Nagata, J. M., Lee, D. J., Ganson, K. T., Toga, A. W., Siegel, S. J., & Jann, K. (2023). Sex differences in regional gray matter density in pre-adolescent binge eating disorder: A voxel-based morphometry study. *Psychological Medicine*, *53*(13), 6077–6089. https://doi.org/10.1017/S0033291722003269

Murray, S. B., Duval, C. J., Balkchyan, A. A., Cabeen, R. P., Nagata, J. M., Toga, A. W., Siegel, S. J., & Jann, K. (2022). Regional gray matter abnormalities in pre-adolescent binge eating disorder: A voxel-based morphometry study. *Psychiatry Research*, *310*, 114473. https://doi.org/10.1016/j.psychres.2022.114473

Murray, S. B., Zhang, R., Duval, C. J., Nagata, J. M., & Jann, K. (2024). Task-evoked neural activity during reward anticipation and inhibitory control in preadolescent binge eating disorder. *Journal of Adolescent Health*, *74*(5), 958–963. https://doi.org/10.1016/j.jadohealth.2023.12.021

Newman, B. T., Patrie, J. T., & Druzgal, T. J. (2023). An intracellular isotropic diffusion signal is positively associated with pubertal development in white matter. *Developmental Cognitive Neuroscience*, *63*, 101301. https://doi.org/10.1016/j.dcn.2023.101301

Nguyen, M. V. H., Xu, Y., Vaughn, K. A., & Hernandez, A. E. (2024). Subcortical and cerebellar volume differences in bilingual and monolingual children: An ABCD study. *Developmental Cognitive Neuroscience*, *65*, 101334. https://doi.org/10.1016/j.dcn.2023.101334

Nishat, E., Stojanovski, S., Scratch, S. E., Ameis, S. H., & Wheeler, A. L. (2023). Premature white matter microstructure in female children with a history of concussion. *Developmental Cognitive Neuroscience*, *62*, 101275. https://doi.org/10.1016/j.dcn.2023.101275

Niu, L., Sheffield, P., & Li, Y. (2023). Pubertal timing, neighborhood income, and mental health in boys and girls: Findings from the Adolescent Brain Cognitive Development study. *Social Science & Medicine*, *334*, 116220. https://doi.org/10.1016/j.socscimed.2023.116220

Olfson, M., Wall, M. M., Wang, S., & Blanco, C. (2023). Prevalence and correlates of mental disorders in children aged 9 and 10 years: Results from the ABCD study. *Journal of the American Academy of Child & Adolescent Psychiatry*. https://doi.org/10.1016/j.jaac.2023.04.005

Owens, M. M., Allgaier, N., Hahn, S., Yuan, D., Albaugh, M., Adise, S., Chaarani, B., Ortigara, J., Juliano, A., Potter, A., & Garavan, H. (2021). Multimethod investigation of the neurobiological basis of ADHD symptomatology in children aged 9-10: Baseline data from the ABCD study. *Translational Psychiatry*, *11*(1), 64. https://doi.org/10.1038/s41398-020-01192-8

Owens, M. M., Hahn, S., Allgaier, N., MacKillop, J., Albaugh, M., Yuan, D., Juliano, A., Potter, A., & Garavan, H. (2022). One-year predictions of delayed reward discounting in the Adolescent Brain Cognitive Development Study. *Experimental and Clinical Psychopharmacology*, *30*(6), 928–946. https://doi.org/10.1037/pha0000532

Owens, M. M., Potter, A., Hyatt, C. S., Albaugh, M., Thompson, W. K., Jernigan, T., Yuan, D., Hahn, S., Allgaier, N., & Garavan, H. (2021). Recalibrating expectations about effect size: A multi-method survey of effect sizes in the ABCD study. *PLOS ONE*, *16*(9), e0257535. https://doi.org/10.1371/journal.pone.0257535

Pagliaccio, D., Alqueza, K. L., Marsh, R., & Auerbach, R. P. (2020). Brain volume abnormalities in youth at high risk for depression: Adolescent Brain and Cognitive Development Study. *Journal of the American Academy of Child & Adolescent Psychiatry*, *59*(10), 1178–1188. https://doi.org/10.1016/j.jaac.2019.09.032

Pagliaccio, D., Durham, K., Fitzgerald, K. D., & Marsh, R. (2021). Obsessive-compulsive symptoms among children in the Adolescent Brain and Cognitive Development Study: Clinical, cognitive, and brain connectivity correlates. *Biological Psychiatry: Cognitive Neuroscience and Neuroimaging*, *6*(4), 399–409. https://doi.org/10.1016/j.bpsc.2020.10.019

Palmer, C. E., Sheth, C., Marshall, A. T., Adise, S., Baker, F. C., Chang, L., Clark, D. B., Coronado, C., Dagher, R. K., Diaz, V., Dowling, G. J., Gonzalez, M. R., Haist, F., Herting, M. M., Huber, R. S., Jernigan, T. L., LeBlanc, K., Lee, K., Lisdahl, K. M., … Yurgelun-Todd, D. (2021). A comprehensive overview of the physical health of the Adolescent Brain Cognitive Development Study cohort at baseline. *Frontiers in Pediatrics*, *9*. https://doi.org/10.3389/fped.2021.734184

Parker, A. J., Walker, J. C., Jordan, L. S., Takarae, Y., Wiggins, J. L., & Dougherty, L. R. (2024). Neural mechanisms of inhibitory control in preadolescent irritability: Insights from the ABCD study. *Biological Psychology*, *192*, 108856. https://doi.org/10.1016/j.biopsycho.2024.108856

Parker, A. J., Walker, J. C., Takarae, Y., Dougherty, L. R., & Wiggins, J. L. (2025). Neural mechanisms of reward processing in preadolescent irritability: Insights from the ABCD study. *Journal of Affective Disorders*, *370*, 286–298. https://doi.org/10.1016/j.jad.2024.10.124

Paul, S. E., Colbert, S. M. C., Gorelik, A. J., Johnson, E. C., Hatoum, A. S., Baranger, D. A. A., Hansen, I. S., Nagella, I., Blaydon, L., Hornstein, A., Elsayed, N. M., Barch, D. M., Bogdan, R., & Karcher, N. R. (2024). A phenome-wide association study of cross-disorder genetic liability in youth genetically similar to individuals from European reference populations. *Nature Mental Health*, *2*(11), 1327–1341. https://doi.org/10.1038/s44220-024-00313-2

Peltz, J., Zhang, L., Sasser, J., Oshri, A., & Doane, L. D. (2024). The influence of pubertal development on early adolescent sleep and changes in family functioning. *Journal of Youth and Adolescence*, *53*(2), 459–471. https://doi.org/10.1007/s10964-023-01882-8

Petrican, R., Miles, S., Rudd, L., Wasiewska, W., Graham, K. S., & Lawrence, A. D. (2021). Pubertal timing and functional neurodevelopmental alterations independently mediate the effect of family conflict on adolescent psychopathology. *Developmental Cognitive Neuroscience*, *52*, 101032. https://doi.org/10.1016/j.dcn.2021.101032

Petrie, D. J., Meeks, K. D., Fisher, Z. F., & Geier, C. F. (2024). Associations between somatomotor-putamen resting state connectivity and obsessive-compulsive symptoms vary as a function of stress during early adolescence: Data from the ABCD study. *Brain Research Bulletin*, *210*, 110934. https://doi.org/10.1016/j.brainresbull.2024.110934

Potter, A., Dube, S., Allgaier, N., Loso, H., Ivanova, M., Barrios, L. C., Bookheimer, S., Chaarani, B., Dumas, J., Feldstein‐Ewing, S., Freedman, E. G., Garavan, H., Hoffman, E., McGlade, E., Robin, L., & Johns, M. M. (2021). Early adolescent gender diversity and mental health in the Adolescent Brain Cognitive Development study. *Journal of Child Psychology and Psychiatry*, *62*(2), 171–179. https://doi.org/10.1111/jcpp.13248

Puga, T. B., Dai, H. D., Wang, Y., & Theye, E. (2024). Maternal tobacco use during pregnancy and child neurocognitive development. *JAMA Network Open*, *7*(2), e2355952. https://doi.org/10.1001/jamanetworkopen.2023.55952

Puga, T. B., Doucet, G. E., Thiel, G. E., Theye, E., & Dai, H. D. (2024). Prenatal tobacco exposure, brain subcortical volumes, and gray-white matter contrast. *JAMA Network Open*, *7*(12), e2451786. https://doi.org/10.1001/jamanetworkopen.2024.51786

Rajagopalan, V., Hsu, E., & Luo, S. (2025). Breastfeeding duration and brain-body development in 9–10-year-olds: Modulating effect of socioeconomic levels. *Pediatric Research*, *97*(1), 378–386. https://doi.org/10.1038/s41390-024-03330-0

Rapuano, K. M., Berrian, N., Baskin-Sommers, A., Décarie-Spain, L., Sharma, S., Fulton, S., Casey, B. J., & Watts, R. (2022). Longitudinal evidence of a vicious cycle between nucleus accumbens microstructure and childhood weight gain. *Journal of Adolescent Health*, *70*(6), 961–969. https://doi.org/10.1016/j.jadohealth.2022.01.002

Rapuano, K. M., Laurent, J. S., Hagler, D. J., Hatton, S. N., Thompson, W. K., Jernigan, T. L., Dale, A. M., Casey, B. J., & Watts, R. (2020). Nucleus accumbens cytoarchitecture predicts weight gain in children. *Proceedings of the National Academy of Sciences*, *117*(43), 26977–26984. https://doi.org/10.1073/pnas.2007918117

Roberts, C., Sahakian, B. J., Chen, S., Sallie, S. N., Walker, C., White, S. R., Weber, J., Skandali, N., Robbins, T. W., & Murray, G. K. (2023). Impact and centrality of attention dysregulation on cognition, anxiety, and low mood in adolescents. *Scientific Reports*, *13*(1), 9106. https://doi.org/10.1038/s41598-023-34399-y

Ronderos, J., Zuk, J., Hernandez, A. E., & Vaughn, K. A. (2024). Large‐scale investigation of white matter structural differences in bilingual and monolingual children: An Adolescent Brain Cognitive Development data study. *Human Brain Mapping*, *45*(2). https://doi.org/10.1002/hbm.26608

Rose, L., Listyg, B., Owens, M. M., Hyatt, C. S., Carter, N. T., Carter, D. R., Lynam, D. R., & Miller, J. D. (2024). Testing whether the relations between sex and psychopathology are accounted for by structural morphometry in ABCD. *Journal of Psychopathology and Clinical Science*, *133*(3), 223–234. https://doi.org/10.1037/abn0000892

Roy, E., Van Rinsveld, A., Nedelec, P., Richie-Halford, A., Rauschecker, A. M., Sugrue, L. P., Rokem, A., McCandliss, B. D., & Yeatman, J. D. (2024). Differences in educational opportunity predict white matter development. *Developmental Cognitive Neuroscience*, *67*, 101386. https://doi.org/10.1016/j.dcn.2024.101386

Sakib, M. N., Best, J. R., & Hall, P. A. (2023). Bidirectional associations between adiposity and cognitive function and mediation by brain morphology in the ABCD Study. *JAMA Network Open*, *6*(2), e2255631. https://doi.org/10.1001/jamanetworkopen.2022.55631

Santos, J. P. L., Versace, A., Ladouceur, C. D., & Soehner, A. M. (2025). The impact of sleep problems during late childhood on internalizing problems in early-mid adolescence. *Behavioral Sleep Medicine*, *23*(1), 31–43. https://doi.org/10.1080/15402002.2024.2401471

Senger-Carpenter, T., Seng, J., Herrenkohl, T. I., Marriott, D., Chen, B., & Voepel-Lewis, T. (2024). Applying life history theory to understand earlier onset of puberty: An Adolescent Brain Cognitive Development cohort analysis. *Journal of Adolescent Health*, *74*(4), 682–688. https://doi.org/10.1016/j.jadohealth.2023.08.013

Senger-Carpenter, T., Seng, J., Marriott, D., Herrenkohl, T. I., Scott, E. L., Chen, B., & Voepel-Lewis, T. (2025). Family adversity and co-occurring pain, psychological, and somatic symptom trajectories from late childhood through early adolescence. *Social Science & Medicine*, *366*, 117650. https://doi.org/10.1016/j.socscimed.2024.117650

Serio, B., Kohler, R., Ye, F., Lichenstein, S. D., & Yip, S. W. (2022). A multidimensional approach to understanding the emergence of sex differences in internalizing symptoms in adolescence. *Developmental Cognitive Neuroscience*, *58*, 101182. https://doi.org/10.1016/j.dcn.2022.101182

Sewaybricker, L. E., Kee, S., Melhorn, S. J., & Schur, E. A. (2021). Greater radiologic evidence of hypothalamic gliosis predicts adiposity gain in children at risk for obesity. *Obesity*, *29*(11), 1770–1779. https://doi.org/10.1002/oby.23286

Shen, C., Luo, Q., Chamberlain, S. R., Morgan, S., Romero-Garcia, R., Du, J., Zhao, X., Touchette, É., Montplaisir, J., Vitaro, F., Boivin, M., Tremblay, R. E., Zhao, X.-M., Robaey, P., Feng, J., & Sahakian, B. J. (2020). What is the link between Attention-Deficit/Hyperactivity Disorder and sleep disturbance? A multimodal examination of longitudinal relationships and brain structure using large-scale population-based cohorts. *Biological Psychiatry*, *88*(6), 459–469. https://doi.org/10.1016/j.biopsych.2020.03.010

Shen, C., Rolls, E. T., Xiang, S., Langley, C., Sahakian, B. J., Cheng, W., & Feng, J. (2023). Brain and molecular mechanisms underlying the nonlinear association between close friendships, mental health, and cognition in children. *ELife*, *12*. https://doi.org/10.7554/eLife.84072

Sisk, L. M., Rapuano, K. M., Conley, M. I., Greene, A. S., Horien, C., Rosenberg, M. D., Scheinost, D., Constable, R. T., Glatt, C. E., Casey, B. J., & Gee, D. G. (2022). Genetic variation in endocannabinoid signaling is associated with differential network‐level functional connectivity in youth. *Journal of Neuroscience Research*, *100*(3), 731–743. https://doi.org/10.1002/jnr.24946

Smolker, H. R., Reid, C. E., Friedman, N. P., & Banich, M. T. (2024). The association between exposure to fine particulate air pollution and the trajectory of internalizing and externalizing behaviors during late childhood and early adolescence: Evidence from the Adolescent Brain Cognitive Development (ABCD) Study. *Environmental Health Perspectives*, *132*(8). https://doi.org/10.1289/EHP13427

Stanley, E. A. M., Wilms, M., Mouches, P., & Forkert, N. D. (2022). Fairness-related performance and explainability effects in deep learning models for brain image analysis. *Journal of Medical Imaging*, *9*(06). https://doi.org/10.1117/1.JMI.9.6.061102

Steward, T., Jann, K., & Murray, S. B. (2024). Distinct functional connectivity phenotypes in preadolescent children with binge eating disorder by BMI status. *Obesity*, *32*(11), 2082–2086. https://doi.org/10.1002/oby.24145

Stinson, E. A., Sullivan, R. M., Navarro, G. Y., Wallace, A. L., Larson, C. L., & Lisdahl, K. M. (2024). Childhood adversity is associated with reduced BOLD response in inhibitory control regions amongst preadolescents from the ABCD study. *Developmental Cognitive Neuroscience*, *67*, 101378. https://doi.org/10.1016/j.dcn.2024.101378

Sun, Y.-J., Sahakian, B. J., Langley, C., Yang, A., Jiang, Y., Kang, J., Zhao, X., Li, C., Cheng, W., & Feng, J. (2024). Early-initiated childhood reading for pleasure: Associations with better cognitive performance, mental well-being and brain structure in young adolescence. *Psychological Medicine*, *54*(2), 359–373. https://doi.org/10.1017/S0033291723001381

Tandberg, A. D., Dahl, A., Norbom, L. B., Westlye, L. T., Ystrom, E., Tamnes, C. K., & Eilertsen, E. M. (2024). Individual differences in internalizing symptoms in late childhood: A variance decomposition into cortical thickness, genetic and environmental differences. *Developmental Science*, *27*(6). https://doi.org/10.1111/desc.13537

Thijssen, S., Collins, P. F., & Luciana, M. (2020). Pubertal development mediates the association between family environment and brain structure and function in childhood. *Development and Psychopathology*, *32*(2), 687–702. https://doi.org/10.1017/S0954579419000580

Thijssen, S., Collins, P. F., & Luciana, M. (2022). Does pubertal stage mediate the association between family environment and structure and function of the amygdala-mPFC circuit? A replication study of the longitudinal ABCD cohort. *Developmental Cognitive Neuroscience*, *56*, 101120. https://doi.org/10.1016/j.dcn.2022.101120

Thomas, E., Juliano, A., Owens, M., Cupertino, R. B., Mackey, S., Hermosillo, R., Miranda-Dominguez, O., Conan, G., Ahmed, M., Fair, D. A., Graham, A. M., Goode, N. J., Kandjoze, U. P., Potter, A., Garavan, H., & Albaugh, M. D. (2024). Amygdala connectivity is associated with withdrawn/depressed behavior in a large sample of children from the Adolescent Brain Cognitive Development (ABCD) Study®. *Psychiatry Research: Neuroimaging*, *344*, 111877. https://doi.org/10.1016/j.pscychresns.2024.111877

Thomas, S. A., Ryan, S. K., & Gilman, J. (2023). Resting state network connectivity is associated with cognitive flexibility performance in youth in the Adolescent Brain Cognitive Development Study. *Neuropsychologia*, *191*, 108708. https://doi.org/10.1016/j.neuropsychologia.2023.108708

Torgerson, C., Ahmadi, H., Choupan, J., Fan, C. C., Blosnich, J. R., & Herting, M. M. (2024). Sex, gender diversity, and brain structure in early adolescence. *Human Brain Mapping*, *45*(5). https://doi.org/10.1002/hbm.26671

van Dijk, M. T., Tartt, A. N., Murphy, E., Gameroff, M. J., Semanek, D., Cha, J., Weissman, M. M., Posner, J., & Talati, A. (2024). Subcortical volumes in offspring with a multigenerational family history of depression – A study across two cohorts. *Journal of Affective Disorders*, *363*, 192–197. https://doi.org/10.1016/j.jad.2024.07.107

Vaughn, K. A., Nguyen, M. V. H., Ronderos, J., & Hernandez, A. E. (2021). Cortical thickness in bilingual and monolingual children: Relationships to language use and language skill. *NeuroImage*, *243*, 118560. https://doi.org/10.1016/j.neuroimage.2021.118560

Vijayakumar, N., Whittle, S., & Silk, T. J. (2023). Corticolimbic connectivity mediates the relationship between pubertal timing and mental health problems. *Psychological Medicine*, *53*(16), 7655–7665. https://doi.org/10.1017/S0033291723001472

Voepel-Lewis, T., Seng, J. S., Chen, B., & Scott, E. L. (2021). A high psychological and somatic symptom profile and family health factors predict new or persistent pain during early adolescence. *The Clinical Journal of Pain*, *37*(2), 86–93. https://doi.org/10.1097/AJP.0000000000000896

Voepel-Lewis, T., Senger-Carpenter, T., Chen, B., Seng, J., Cofield, C., Ploutz-Snyder, R., & Scott, E. L. (2023). Associations of co-occurring symptom trajectories with sex, race, ethnicity, and health care utilization in children. *JAMA Network Open*, *6*(5), e2314135. https://doi.org/10.1001/jamanetworkopen.2023.14135

Walsh, J. J., Barnes, J. D., Tremblay, M. S., & Chaput, J.-P. (2020). Associations between duration and type of electronic screen use and cognition in US children. *Computers in Human Behavior*, *108*, 106312. https://doi.org/10.1016/j.chb.2020.106312

Wang, H., Rolls, E. T., Du, X., Du, J., Yang, D., Li, J., Li, F., Cheng, W., & Feng, J. (2020). Severe nausea and vomiting in pregnancy: Psychiatric and cognitive problems and brain structure in children. *BMC Medicine*, *18*(1), 228. https://doi.org/10.1186/s12916-020-01701-y

Whitmore, L. B., Weston, S. J., & Mills, K. L. (2023). BrainAGE as a measure of maturation during early adolescence. *Imaging Neuroscience*, *1*, 1–21. https://doi.org/10.1162/imag_a_00037

Wiglesworth, A., Falke, C. A., Fiecas, M., Luciana, M., Cullen, K. R., & Klimes-Dougan, B. (2023). Brain signatures in children who contemplate suicide: Learning from the large-scale ABCD Study. *Psychological Medicine*, *53*(5), 2164–2173. https://doi.org/10.1017/S0033291721004074

Wiglesworth, A., Fiecas, M. B., Xu, M., Neher, A. T., Padilla, L., Carosella, K. A., Roediger, D. J., Mueller, B. A., Luciana, M., Klimes-Dougan, B., & Cullen, K. R. (2023). Sex and age variations in the impact of puberty on cortical thickness and associations with internalizing symptoms and suicidal ideation in early adolescence. *Developmental Cognitive Neuroscience*, *59*, 101195. https://doi.org/10.1016/j.dcn.2022.101195

Wu, X., Palaniyappan, L., Yu, G., Zhang, K., Seidlitz, J., Liu, Z., Kong, X., Schumann, G., Feng, J., Sahakian, B. J., Robbins, T. W., Bullmore, E., & Zhang, J. (2023). Morphometric dis-similarity between cortical and subcortical areas underlies cognitive function and psychiatric symptomatology: A preadolescence study from ABCD. *Molecular Psychiatry*, *28*(3), 1146–1158. https://doi.org/10.1038/s41380-022-01896-x

Wu, X., Yu, G., Zhang, K., Feng, J., Zhang, J., Sahakian, B. J., & Robbins, T. W. (2022). Symptom-based profiling and multimodal neuroimaging of a large preteenage population identifies distinct Obsessive-Compulsive Disorder–like subtypes with neurocognitive differences. *Biological Psychiatry: Cognitive Neuroscience and Neuroimaging*, *7*(11), 1078–1089. https://doi.org/10.1016/j.bpsc.2021.06.011

Yamashita, M., Shou, Q., & Mizuno, Y. (2024a). Association of chronotype with language and episodic memory processing in children: Implications for brain structure. *Frontiers in Integrative Neuroscience*, *18*. https://doi.org/10.3389/fnint.2024.1437585

Yamashita, M., Shou, Q., & Mizuno, Y. (2024b). Unsupervised machine learning for identifying attention-deficit/hyperactivity disorder subtypes based on cognitive function and their implications for brain structure. *Psychological Medicine*, *54*(14), 3917–3929. https://doi.org/10.1017/S0033291724002368

Yan, J., Bai, H., Sun, Y., Sun, X., Hu, Z., Liu, B., He, C., & Zhang, X. (2024). Frontoparietal response to working memory load mediates the association between sleep duration and cognitive function in children. *Brain Sciences*, *14*(7), 706. https://doi.org/10.3390/brainsci14070706

Yang, A., Rolls, E. T., Dong, G., Du, J., Li, Y., Feng, J., Cheng, W., & Zhao, X.-M. (2022). Longer screen time utilization is associated with the polygenic risk for Attention-Deficit/Hyperactivity Disorder with mediation by brain white matter microstructure. *EBioMedicine*, *80*, 104039. https://doi.org/10.1016/j.ebiom.2022.104039

Yang, F. N., Liu, T. T., & Wang, Z. (2022). Functional connectome mediates the association between sleep disturbance and mental health in preadolescence: A longitudinal mediation study. *Human Brain Mapping*, *43*(6), 2041–2050. https://doi.org/10.1002/hbm.25772

Yang, F. N., Liu, T. T., & Wang, Z. (2023). Corticostriatal connectivity mediates the reciprocal relationship between parent‐reported sleep duration and impulsivity in early adolescents. *Journal of Child Psychology and Psychiatry*, *64*(11), 1545–1554. https://doi.org/10.1111/jcpp.13843

Yang, F. N., Xie, W., & Wang, Z. (2022). Effects of sleep duration on neurocognitive development in early adolescents in the USA: A propensity score matched, longitudinal, observational study. *The Lancet Child & Adolescent Health*, *6*(10), 705–712. https://doi.org/10.1016/S2352-4642(22)00188-2

Zhang, L., Sasser, J., Doane, L. D., Peltz, J., & Oshri, A. (2024). Latent profiles of sleep patterns in early adolescence: Associations with behavioral health risk. *Journal of Adolescent Health*, *74*(1), 177–185. https://doi.org/10.1016/j.jadohealth.2023.08.021

Zhang, Ru, Murray, S. B., Duval, C. J., Wang, D. J. J., & Jann, K. (2024). Functional connectivity and complexity analyses of resting-state fMRI in pre-adolescents demonstrating the behavioral symptoms of ADHD. *Psychiatry Research*, *334*, 115794. https://doi.org/10.1016/j.psychres.2024.115794

Zhang, Rui, Manza, P., & Volkow, N. D. (2022). Prenatal caffeine exposure: Association with neurodevelopmental outcomes in 9‐ to 11‐year‐old children. *Journal of Child Psychology and Psychiatry*, *63*(5), 563–578. https://doi.org/10.1111/jcpp.13495

Zhang, X., Sun, Y., Wang, M., Zhao, Y., Yan, J., Xiao, Q., Bai, H., Yao, Z., Chen, Y., Zhang, Z., Hu, Z., He, C., & Liu, B. (2025). Multifactorial influences on childhood insomnia: Genetic, socioeconomic, brain development and psychopathology insights. *Journal of Affective Disorders*, *372*, 296–305. https://doi.org/10.1016/j.jad.2024.12.031

Zhao, Y., Paulus, M. P., Tapert, S. F., Bagot, K. S., Constable, R. T., Yaggi, H. K., Redeker, N. S., & Potenza, M. N. (2024). Screen time, sleep, brain structural neurobiology, and sequential associations with child and adolescent psychopathology: Insights from the ABCD study. *Journal of Behavioral Addictions*, *13*(2), 542–553. https://doi.org/10.1556/2006.2024.00016

Zhi, D., Jiang, R., Pearlson, G., Fu, Z., Qi, S., Yan, W., Feng, A., Xu, M., Calhoun, V., & Sui, J. (2024). Triple interactions between the environment, brain, and behavior in children: An ABCD study. *Biological Psychiatry*, *95*(9), 828–838. https://doi.org/10.1016/j.biopsych.2023.12.019

Zhou, Q., Zhao, X., Chen, J., Xu, J., Yang, A., Xiong, Y., Yin, X., Zhao, X.-M., & Li, X. (2025). Association between twin status with cognitive, behavioral development and brain structure in early adolescence: a retrospective cohort analysis based on the Adolescent Brain Cognitive Development Study. *European Child & Adolescent Psychiatry*, *34*(2), 695–707. https://doi.org/10.1007/s00787-024-02515-6

Zhou, Q., Zhao, X., Chen, J., Yang, A., Zhao, X.-M., & Li, X. (2024). Association of birth weight with neuropsychological functioning in early adolescence: A retrospective cohort study. *Psychiatry Research*, *342*, 116183. https://doi.org/10.1016/j.psychres.2024.116183

Zink, J., O’Connor, S. G., Blachman-Demner, D. R., Wolff-Hughes, D. L., & Berrigan, D. (2024). Examining the bidirectional associations between sleep duration, screen time, and internalizing symptoms in the ABCD study. *Journal of Adolescent Health*, *74*(3), 496–503. https://doi.org/10.1016/j.jadohealth.2023.09.001

**Appendix S4: Sibling exclusion criteria in the empirical comparison**

Analyses including only one sibling typically used the first sibling enrolled. If two siblings were enrolled on the same day, the one whose ID is first alphabetically was included; this was usually the older sibling.
